# Exploring O_2_ Diffusion in A-Type Cytochrome *c* Oxidases: Molecular Dynamics Simulations Uncover Two Alternative Channels towards the Binuclear Site

**DOI:** 10.1371/journal.pcbi.1004010

**Published:** 2014-12-04

**Authors:** A. Sofia F. Oliveira, João M. Damas, António M. Baptista, Cláudio M. Soares

**Affiliations:** ITQB - Instituto de Tecnologia Química e Biológica António Xavier, Universidade Nova de Lisboa, Oeiras, Portugal; Max Planck Institute for Biophysical Chemistry, Germany

## Abstract

Cytochrome *c* oxidases (C*c*oxs) are the terminal enzymes of the respiratory chain in mitochondria and most bacteria. These enzymes couple dioxygen (O_2_) reduction to the generation of a transmembrane electrochemical proton gradient. Despite decades of research and the availability of a large amount of structural and biochemical data available for the A-type C*c*ox family, little is known about the channel(s) used by O_2_ to travel from the solvent/membrane to the heme *a_3_*-Cu_B_ binuclear center (BNC). Moreover, the identification of all possible O_2_ channels as well as the atomic details of O_2_ diffusion is essential for the understanding of the working mechanisms of the A-type C*c*ox. In this work, we determined the O_2_ distribution within C*c*ox from *Rhodobacter sphaeroides*, in the fully reduced state, in order to identify and characterize all the putative O_2_ channels leading towards the BNC. For that, we use an integrated strategy combining atomistic molecular dynamics (MD) simulations (with and without explicit O_2_ molecules) and implicit ligand sampling (ILS) calculations. Based on the 3D free energy map for O_2_ inside C*c*ox, three channels were identified, all starting in the membrane hydrophobic region and connecting the surface of the protein to the BNC. One of these channels corresponds to the pathway inferred from the X-ray data available, whereas the other two are alternative routes for O_2_ to reach the BNC. Both alternative O_2_ channels start in the membrane spanning region and terminate close to Y288_I_. These channels are a combination of multiple transiently interconnected hydrophobic cavities, whose opening and closure is regulated by the thermal fluctuations of the lining residues. Furthermore, our results show that, in this C*c*ox, the most likely (energetically preferred) routes for O_2_ to reach the BNC are the alternative channels, rather than the X-ray inferred pathway.

## Author Summary

Cytochrome *c* oxidases (C*c*oxs), the terminal enzymes of the respiratory electron transport chain in eukaryotes and many prokaryotes, are key enzymes in aerobic respiration. These proteins couple the reduction of molecular dioxygen to water with the creation of a transmembrane electrochemical proton gradient. Over the last decades, most of the C*c*oxs research focused on the mechanisms and energetics of reduction and/or proton pumping, and little emphasis has been given to the pathways used by dioxygen to reach the binuclear center, where dioxygen reduction takes place. In particular, the existence and the characteristics of the channel(s) used by O_2_ to travel from the solvent/membrane to the binuclear site are still unclear. In this work, we combine all-atom molecular dynamics simulations and implicit ligand sampling calculations in order to identify and characterize the O_2_ delivery channels in the C*c*ox from *Rhodobacter sphaeroides*. Altogether, our results suggest that, in this C*c*ox, O_2_ can diffuse via three well-defined channels that start in membrane region (where O_2_ solubility is higher than in the water). One of these channels corresponds to the pathway inferred from the X-ray data available, whereas the other two are alternative routes for O_2_ to reach the binuclear center.

## Introduction

Cytochrome *c* oxidases (C*c*oxs) are the terminal enzymes of the respiratory chain in eukaryotes and in aerobic prokaryotes (reviewed in [1]). These integral membrane proteins belong to the heme-copper oxidases superfamily and couple dioxygen (O_2_) reduction to the translocation of protons across the membrane. C*c*ox takes up four electrons from cytochrome *c* (*cyt c*) in the positively charged side of the membrane (the inter-membrane space in mitochondria or the periplasm in bacteria) and eight protons from the negatively charged side (eq. 1) [2], [3]:
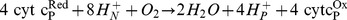
(1)where the subscripts P and N refer to the positive and negative sides of the membrane, respectively.

Four of the eight protons reported in equation 1 are used to reduce one O_2_ molecule and form two water molecules [2], [3], whereas the remaining protons are pumped from the negative to the positive side of the membrane. This overall process contributes to the generation and maintenance of a transmembrane electrochemical proton gradient, which can be further utilized for several energy-requiring processes, such as ATP synthesis [4].

**Figure 1:**
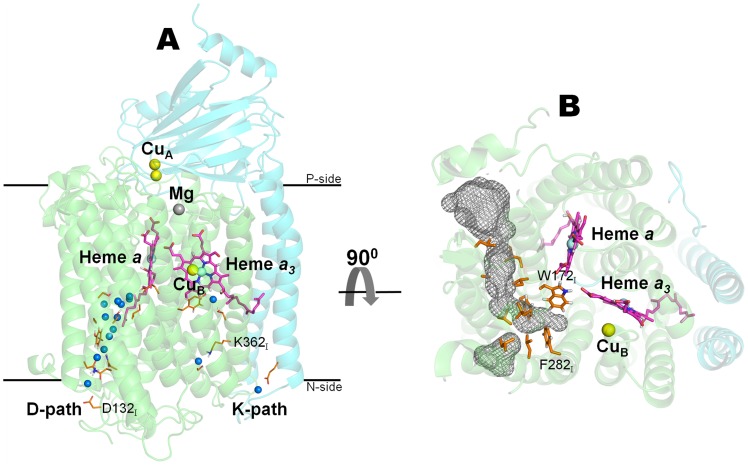
Structure of the membrane bound C*c*ox from *R. sphaeroides* [15] . For simplicity, only subunits I and II are represented. **A**) Architecture of the C*c*ox core functional unit formed by subunits I (colored in green) and II (colored in cyan). The small blue spheres represent the water molecules resolved in the D- and K-pathways in the X-ray structure [15]. During an O_2_ reduction cycle, the electrons are sequentially donated from cyt *c* and delivered to the BNC, via Cu_A_ and heme *a*, like in the scheme: cyt *c* → Cu_A_ → heme *a* → BNC (heme *a_3_*/Cu_B_) → O_2_. **B**) Putative O_2_ channel inferred from the X-ray data available. The grey mesh represents the putative O_2_ channel and it was calculated with the program HOLLOW [92].

Based on structural and phylogenetic analysis, the heme-copper oxidases superfamily is currently divided into three major subfamilies [5]: A, B and C. The main differences between the three families are the pathways and mechanisms of proton transfer/pumping. The A-type C*c*oxs, which are the subject of this work, are widespread through all kingdoms of life [5] and among them are the most thoroughly explored C*c*oxs [3], [6], such as the bovine heart mitochondria, the *Paracoccus* (*P.*) *denitrificans* and the *Rhodobacter (R.) sphaeroides* enzymes. These C*c*oxs contain, in the catalytic subunit (subunit I), a low spin heme *a* and a heterodinuclear center named binuclear center, BNC (Fig. 1A). The BNC is deeply buried in the core of the protein and it is formed by a high-spin heme *a_3_* and a copper ion (Cu_B_). In subunit II, these C*c*oxs contain only one redox center, a binuclear copper center named Cu_A_, which accepts electrons from the soluble cyt *c* and then transfers them to the BNC via heme *a*.

It is believed that protons (both chemical and pumped) are transported from the N-side of the membrane to the BNC via two special proton conducting pathways [3]: the D- and K-pathways (Fig. 1A). A third putative proton-conducting pathway, the H-pathway, was proposed for the mammalian C*c*ox only [6], [7], and it was suggested to be exclusively used for the transfer of the pumped protons [8].

Several high-resolution crystallographic structures of the A-type family are nowadays available in the literature (e.g. mammalian [7], [9]–[11] and bacterial C*c*oxs [12]–[15]) and, based on these structures, it is known that all A-type members share a remarkable structural similarity of the core functional unit formed by subunits I and II (Fig. 1A). Subunit I consists of twelve transmembrane α-helices and contains the BNC and the heme *a* center. Subunit II is formed by a solvent exposed globular β-sheet domain (which functions as a docking surface for cyt *c*) and two transmembrane α-helices. It contains only one redox center, the binuclear copper center (Cu_A_). Moreover, at the interface between subunits I and II, C*c*ox has one Mg^+2^ ion whose function is still not well understood, but it was suggested to be part of the exit pathway for the pumped protons and for water formed in the BNC [16], [17]. Subunit III, although not considered to be part of the core functional unit, is also highly conserved among the A-type subfamily. Nevertheless, its absence significantly increases the probability of suicide inactivation [18], [19] and thereby reduces the catalytic lifespan of C*c*ox (in 600-fold or more) [19].

Based in the X-ray data available (eg. [7], [12], [13]), a putative O_2_ channel for the A-type family was proposed (Fig. 1B). Iwata and co-workers, after pressurizing *R. sphaeroides* C*c*ox crystals with xenon, were able to identify a continuous hydrophobic channel that starts in the membrane region of subunit I [13]. This putative O_2_ channel has two possible entrances that merge together in a region close to the proton-gating residue, E286_I_ (the residues are numbered according to the *R. sphaeroides* C*c*ox sequence and the subscript indicates the subunit number). This pathway presents a constriction point which does not allow the access of O_2_ to the BNC, at least without the occurrence of some conformational change in the protein. Unfortunately, until now, none of the mutagenesis and biochemical studies performed in this channel [20]–[22] was able to clearly demonstrate that it serves as an O_2_ route into the BNC. All the tested mutations were located too close to the BNC [20], [21], which made the interpretation of the results difficult and did not allow to unambiguously distinguish between the structural obstruction of the O_2_ channel and the perturbation of the BNC binding kinetics. However, and contrary to the A-type family, in the B-type family the channel used by O_2_ to reach the BNC is nowadays considered to be well established. The crystallographic studies (with xenon pressurization) performed in the *Thermus* (*T.*) *thermophilus ba_3_* enzyme [23]–[25], lead to the identification of a “Y-shaped” hydrophobic channel that runs from the membrane region towards the BNC. This channel, although located roughly at the same position of the putative O_2_ channel in the A-type C*c*ox, does not possess a constriction point close to the BNC. In the A-type C*c*oxs, the narrowing of the O_2_ channel is mainly caused by two conserved bulky residues (W172_I_ and F282_I_ in *R. sphaeroides* [13]), whereas in the B-type C*c*ox, smaller residues occupy these positions (Y133_I_ and T231_I_ in *T. thermophilus* [23], [24]). The differences between the A- and B-type regarding the O_2_ channel are thought to reflect the different functional environments of each type of C*c*ox.

Although the static crystal structures have been a valuable tool for providing insights into the O_2_ diffusion and for identifying potential O_2_ channels in C*c*ox, the elucidation of the molecular basis of O_2_ diffusion requires the knowledge of the C*c*ox conformational dynamics. Transiently formed cavities and openings inside the protein (frequently regulated by side chain rotation or by water movements) are not visible in the static X-ray structures, but have already been shown to be very relevant for ligand diffusion (see for example [26]). In this context, molecular dynamics (MD) simulation techniques (with sufficient simulation time and conformational sampling) appear as an alternative for studying the dynamic behavior of proteins and to determine their ligand occupation probabilities inside the protein. In the last decade, computational methods have been widely used to study gas migration in a number of proteins and MD simulations have successfully allowed the identification of several alternative routes for ligand diffusion (e.g. hydrogenase [26]–[28], myoglobins [29], [30], oxidases [31], [32] and laccases [33]). Moreover, the combination of MD simulations with Implicit Ligand Sampling (ILS) [29] calculations allows the calculation of the energy cost of transferring any small, apolar molecule (like O_2_ or H_2_) from the solvent to the protein and consequently to compute a 3D free-energy landscape for that specific ligand molecule (e.g [29], [33], [34]).

Over the last decades, most of the C*c*ox research using computational methods focused on the mechanisms and energetics of reduction and/or proton pumping (e.g [17], [35]–[56]). In the A-type C*c*ox, little emphasis has been given to the identification of the routes used by O_2_ to move from the solvent towards the BNC, a question only addressed, to our knowledge, by Hofacker and Schulten [31] and by Farantos and co-workers [31]. In the first work, Hofacker and Schulten [31] used MD simulations to study O_2_ diffusion in the vicinity of the BNC in a bacterial C*c*ox from *P. denitrificans* and in the bovine CcOx enzyme. Their simulations revealed a unique, well-defined O_2_ diffusion channel starting in the membrane-spanning surface of subunit I, close to the interface with subunit III. More recently, Farantos and co-workers [32] have applied the ILS method in order to study the binding of several small gas molecules around the BNC region in the A-type C*c*ox from *P. denitrificans* and in the B-type C*co*x enzyme from *T thermophilus*. From these calculations, the authors were able to identify several cavities around the heme *a_3_* region that are conserved in both the A-type and B-type enzymes. This study is however limited to the BNC region, not including other parts of the protein and, consequently, not allowing the analysis of the whole O_2_ permeation process.

The main objective of this work is to identify the O_2_ channels in the fully reduced C*c*ox from *R. sphaeroides* [15] using a combination of MD simulations (with and without explicit O_2_) and ILS calculations. Our results revealed the existence of three putative O_2_ diffusion channels. One of channels correlates very well with the channel inferred from the X-ray data available, whereas the other two are alternative routes for O_2_ to reach the BNC, and were not observed in the X-ray structures pressurized with xenon. Both alternative channels start in the membrane phase and terminate close to Y288_I_.

## Results/Discussion

In this work, we investigate the diffusion of O_2_ molecules from the solvent to C*c*ox using MD simulations. We started our study by performing simulations with the enzyme (in the fully reduced state) with explicit O_2_ molecules, as described in detail in the Materials and Methods. Although all O_2_ molecules were initially placed randomly in the solvent, as time progresses, the gas enters the membrane and concentrates in the lipid tails region (see S8 Figure in Text S1), similarly to what has been described experimentally [57], [58]. After 100 ns of simulation, 46 O_2_ molecules were internalized in the membrane, which corresponds to more than 50% of the O_2_ placed originally inside the simulation box.

**Figure 2:**
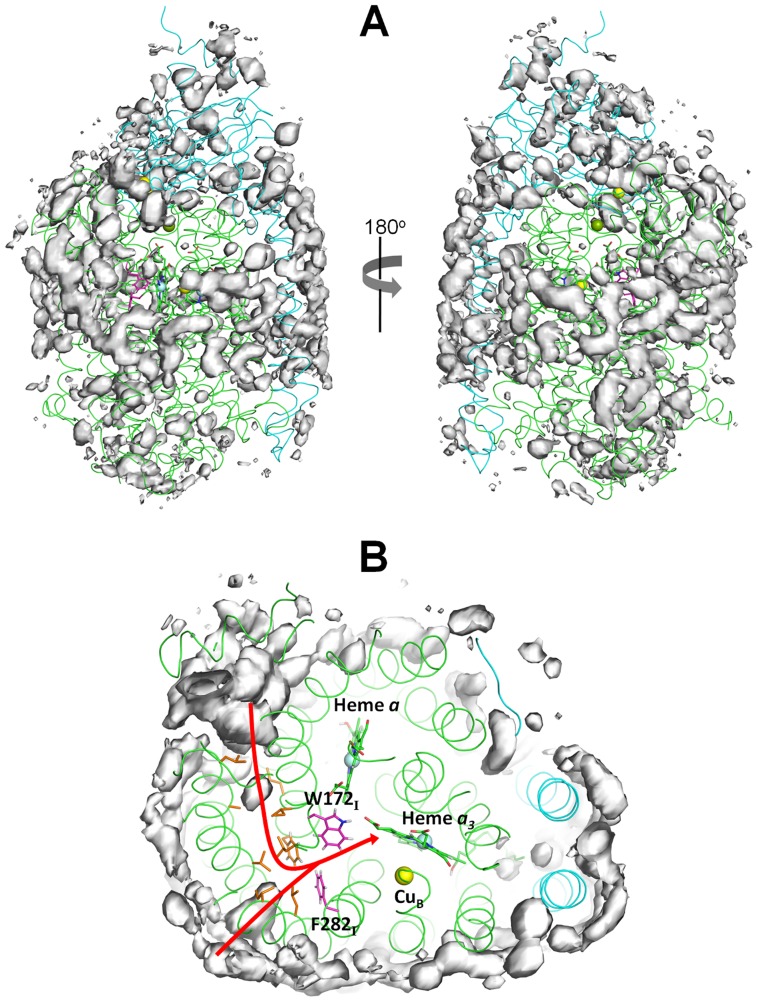
O_2_ probability density maps obtained from all-atom MD simulations. **A**) The probability density contours at 0.00015 Å^−3^ are depicted as a grey surface. The X-ray structure is represented as a ribbon with subunit I colored in green and subunit II in cyan. The hemes are depicted as green sticks. The yellow, light blue and green spheres represent the Cu, Fe (from the heme groups) and the Mg atoms, respectively. The residues forming the putative O_2_ channel inferred from the X-ray structure (according to [13]) are colored in orange and the residues forming the constriction point in this channel are highlighted in magenta. **B**) Zoom image of the putative O_2_ channel inferred from the X-ray structure pressurized with xenon [13]. The red arrow identifies the two possible entrance points for this O_2_ channel.

Furthermore, during the simulation time, some O_2_ molecules move from the membrane into the protein. The number of O_2_ inside C*co*x increased slowly during the first 30 ns of simulation until it stabilizes at ∼8 molecules on average (see S9 Figure in S1 Text). In general, before entering C*c*ox, the O_2_ molecules explore the protein's surface and bind briefly to the cavities and niches formed mainly by hydrophobic residues. However, after 100 ns, none of the internalized O_2_ molecules was able to reach the BNC in any replicate. Nonetheless, and in order to determine which regions of the protein are more populated by the O_2_ molecules during our simulations, we calculated the O_2_ probability density maps [59] over the 100 ns (for all five replicates) and the results are depicted in Fig. 2.

As it can be seen, most of the high-affinity regions are located in the membrane phase (Fig. 2A) and some of them show a good correlation with the putative O_2_ channel inferred from the X-ray data pressurized with xenon [13] (Fig. 2B). This channel starts at the membrane spanning region in direct contact with the hydrophobic tails of the lipids and it is formed predominately by uncharged and aromatic residues. It has a Y shape with the two entrances located between helices 5 and 8 and helices 11 and 13 in subunit I. The side chains of I104_I_, L105_I_, A153_I_, L157_I_, F108_I_, L174_I_, V194_I_, L246_I_, and I250_I_ contribute to form this channel. The two possible entrances merge together into a constriction point located close to E286_I_. This reduction in the diameter of the channel is caused by the phenyl ring of F282_I_ and the indole ring of W172_I_ (see Fig. 2B). It has been suggested that during the O_2_ reduction cycle, some protein rearrangement or side chain rotation (of F282_I_ and/or W172_I_) is required in order to open the constriction point and allow O_2_ access to the BNC [7], [23]. In our O_2_ simulations, the side chains of F282_I_ and W172_I_, although showing some flexibility and being able to slightly change their conformation, did not open an entrance point large enough to allow the passage of O_2_ into the BNC. For this reason, it was impossible to directly observe O_2_ diffusion towards the catalytic site.

Recent computational studies have suggested that the hydration level of the internal hydrophobic cavity located after the constriction point (formed by F282_I_, W172_I_ and E286_I_) could be important for the regulation of proton transfer in C*c*oxs (e.g [55], [56]). It was also suggested that the water distribution inside this cavity is modulated by the protonation of the heme *a_3_* D-propionate and that these hydration changes strongly affect the E286_I_ proton affinity [56]. This hydrophobic cavity bridges between the end of the D-pathway and the BNC region and it has been predicted to contain several water molecules, at least transiently (e.g. [38], [60]–[62]). However, in our simulations, no water molecules were observed in this region and no.direct and visible connection between E286_I_ and the BNC was identified.

Since the simulations with explicit O_2_ were not able to properly sample the diffusion events (probably due to the simulation timescales), and in order to identify possible alternative O_2_ diffusion channels in fully reduced C*c*ox, the ILS method [29] was used. This method uses the protein conformations obtained from a ligand-free MD trajectory and calculates, for all the positions inside the protein, the potential of mean force for placing a small, apolar, low-interacting molecule at that point. The 3D energy map generated in this case represents the Gibbs free energy of moving a O_2_ molecule from water into any place inside the protein (Δ*G*_wat→prot_(O_2_)) and it can be used to infer about the energetically favourable diffusion paths inside a given protein. In these maps, the regions where Δ*G*_wat→prot_(O_2_) is low represent the positions where O_2_ has a high probability of residing, meaning that the O_2_ affinity in that position is high. The ILS method reduces significantly the sampling problems and it takes into account the dynamic conformational changes of the protein and all the transiently formed cavities, which can be combined to form transitory diffusion pathways.

**Figure 3:**
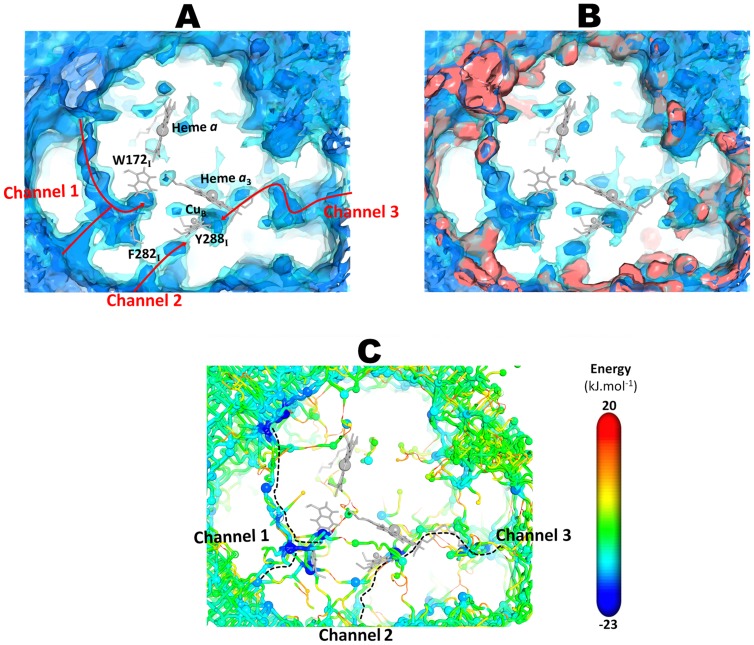
O_2_ free energy landscape obtained from the ILS calculations and lowest free energy pathways obtained from the ILS energy landscape. **A-**O_2_ free energy landscape obtained from the ILS calculations. Two isosurface contours are shown, with free energy levels at -5 (blue inner contour) and 5 (cyan contour) kJ·mol^−1^. **B-** Comparison of the O_2_ free energy landscape obtained from the ILS calculations (blue maps) and high-affinity regions from the O_2_ explicit MD simulations (red maps). The probability density contours at 0.00015 Å^−3^ are represented as a red isosurface. **C-** O_2_ lowest free energy pathways obtained from the ILS energy landscape. The spheres represent the local free energy minima while the tubes connecting the minima represent the pathways. The size of the spheres representing the local energy minima scales linearly along the displayed free energy range (small spheres indicates high free energy while large spheres indicate low free energy). The diameter of the pathways scales linearly with the free energy and thus with the oxygen affinity (thinner radius represent high free energies and low oxygen affinity). The free energy at the minima and along the pathways follows the same color scale. The channel 1 coincides with the channel inferred from the X-ray data [7], [13] whereas the channel 2 and channel 3 are alternative routes to reach the BNC.

From the 3D affinity map for C*c*ox obtained from the ILS calculations (Fig. 3A), we can see that C*c*ox possesses a complex free energy landscape with several possible O_2_ binding cavities, most of them located at the protein surface and that do not directly connect to the BNC. Moreover, the calculated affinity map from ILS not only correlates well with some of the high probability regions found in the explicit O_2_ simulations (see Fig. 3B) but also provides a more complete description of the free energy landscape for O_2_ inside C*c*ox due to the sampling of lower affinity zones, not sufficiently sampled by the normal MD simulations. Indeed, the ILS approach allowed us to identified three major routes (named channel 1, channel 2 and channel 3) with high probability of O_2_ occupancy, interconnecting the protein surface to the BNC. Two of the channels (channel 1 and channel 2) approach the BNC from the subunit I side whereas the third one (channel 3) approaches the BNC from the subunit II side (Fig. 3A). Interestingly all three channels start in the membrane spanning region where the O_2_ concentration is higher than in aqueous phase, which makes physical sense.

Furthermore and in order to identify the energetically preferred routes for O_2_ to access the BNC, we extracted, from the ILS affinity map, the lowest free energy pathways connecting the exterior with the O_2_ high affinity sites (basins in the O_2_ free energy landscape) inside C*c*ox, using the same methodology as Damas *et al* [33] (for details, see the Data Analysis section of Materials and Methods). From the analysis of the O_2_ free energy landscape (Fig. 3C), we observe that there are many energy minima and low free energy pathways connecting the solvent/membrane region to the interior of the protein. Nonetheless, all these low free energy entrance channels converge into three distinct pathways as we approach the BNC.

**Figure 4:**
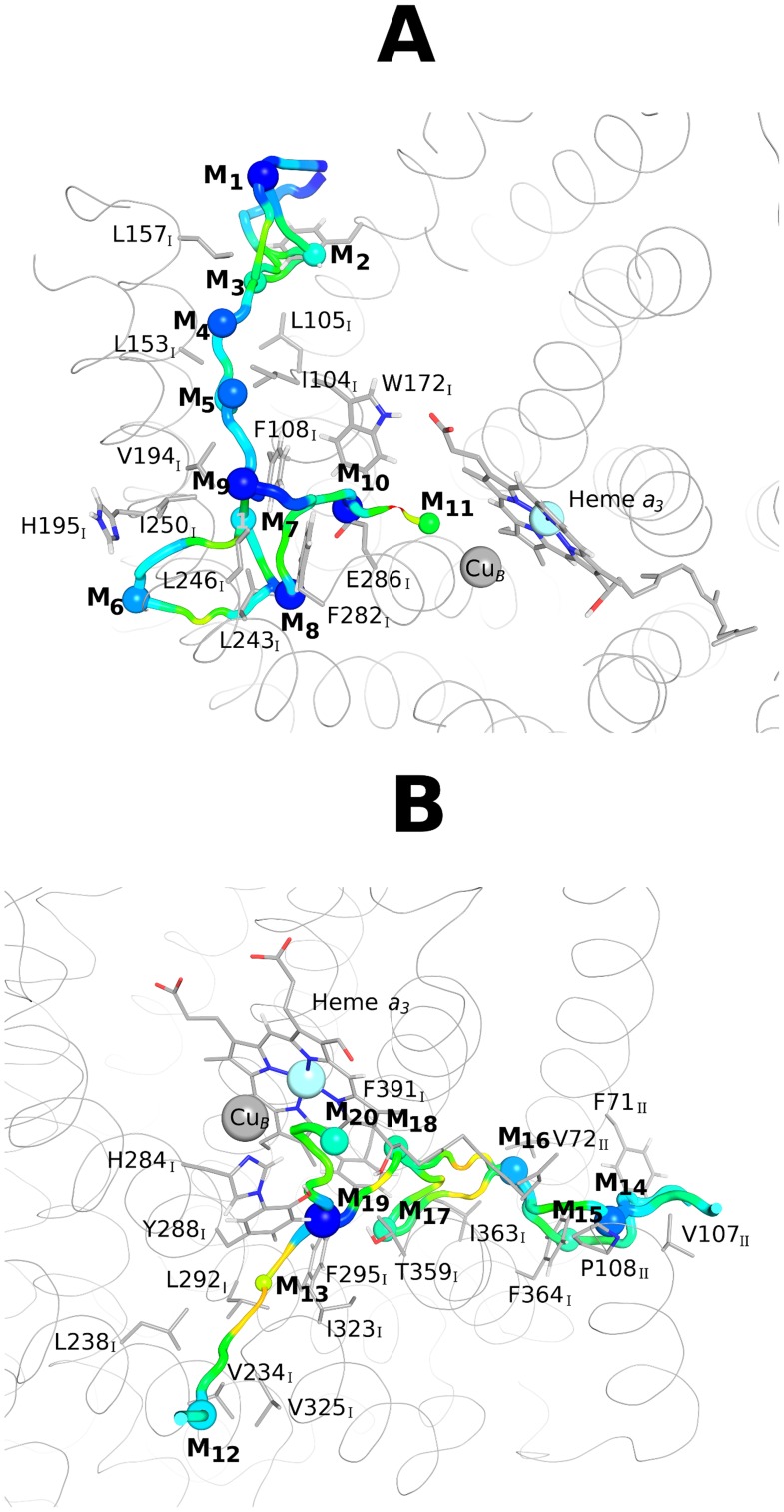
Overview of the O_2_ lowest free energy pathways obtained from the ILS free energy landscape. For legend details related with the representation of the free energy minima and the pathways connecting them, see Fig. 3C. Heme *a_3_* is represented in grey sticks whereas the grey spheres correspond to the Cu atom from the Cu_B_ center and to the Fe atom from heme *a_3_*. The residues forming the O_2_ channels are shown in sticks with a sequence label. **A**- Lowest free energy pathway and free energy minima for the O_2_ channel 1. The free energy values of the represented minima (in kJ·mol^−1^) are: −21.07 (M_1_), −11.79 (M_2_), −10.58 (M_3_), −17.97 (M_4_), −17.53 (M_5_), −16.25 (M_6_), −13.87 (M_7_), −20.07 (M_8_), −20.47 (M_9_), −20.47 (M_10_) and −7.12 (M_11_). **B**- Lowest free energy pathways and minima for the O_2_ channel 2 and channel 3. For the O_2_ channel 2, the values (in kJ·mol^−1^) for the minima are: −13.87 (M_12_), 0.14 (M_13_), −22.77 (M_19_) and −11.03 (M_20_). The free energy values (in kJ·mol^−1^) of the minima forming the O_2_ channel 3 are: −17.24 (M_14_), −10.46 (M_15_), −15.81 (M_16_), −10.40 (M_17_), −10.18 (M_18_), −22.77 (M_19_) and −11.03 (M_20_).

**Figure 5:**
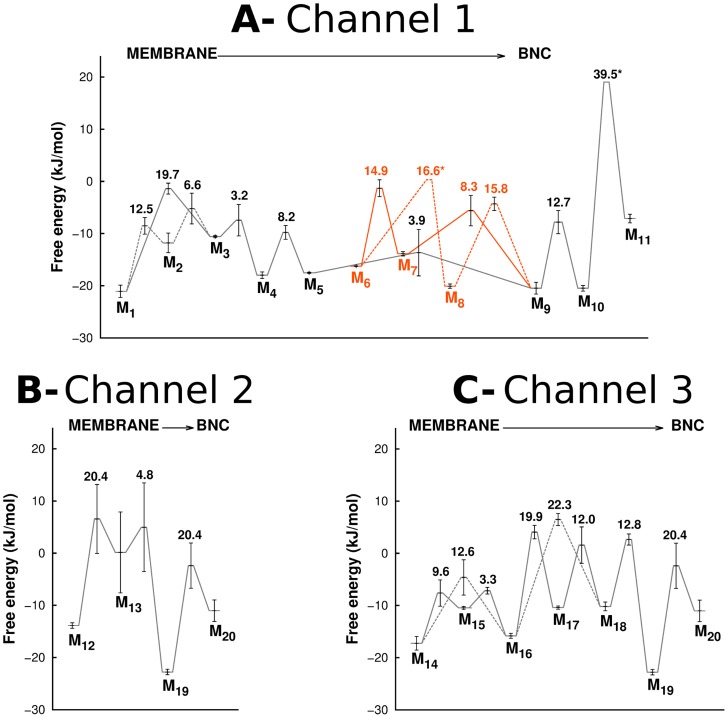
Free energy barriers experienced by O_2_. Free energy barriers experienced by O_2_ when moving from the membrane region to the BNC, along Channel 1 (A), Channel 2 (B) and Channel 3 (C). For details related to the errors calculation see the data analysis section of the Materials and Methods. The “*” in the fig. indicates the transitions for which the errors could not be calculated. In Fig. A, the black lines correspond to Channel 1, which starts between helices 5 and 8 of subunit I, whereas the orange lines correspond to the second entrance point located between helices 11 and 13 of subunit I. In A and C, the dashed lines correspond to alternative routes for O_2_ inside the same channel (for example in Fig. C, O_2_ can move directly from M_14_ to M_16_, or it can go from M_14_ to M_15_ and only then to M_16_). The numbers inside the plots on top of the transition states indicate the free energy barriers experienced by O_2_ when moving from the membrane in the direction of the BNC (i.e. between the different minima on their immediate left and the transition states in question).

The O_2_ channel 1 approaches the BNC from the subunit I side and corresponds to the channel inferred from the X-ray data (for *R. sphaeroides* [13] or for bovine [7]). This pathway has two entry points that are fused together in a free energy minimum located in the constriction point just before the BNC (M_10_ in Fig. 4A). The free energy profile for this pathway (Fig. 4A) is characterized by a very high permeation free energy barrier in the constriction point (associated to the bulky side chain of W172_I_) and three deep local minima (M_8_, M_9_ and M_10_ in Fig. 4A) located between W172_I_, F282_I_ and E286_I_ for M_10_, L243_I_, F282_I_ and L283_I_ for M_8_, and I250_I_, V194_I_ and F108_I_ for M_9_. The local minima observed just below the C*c*ox surface (M_1_ and M_6_) can probably act as scavengers for the O_2_ freely diffusing in the membrane and help to create an O_2_ reservoir inside the protein. In this pathway, W172_I_ and E286_I_ seem to act as the gateway residues that control O_2_ access to the catalytic site. Nevertheless, the passage from the constriction point to the BNC (moving from M_10_ to M_11_ in Fig. 4A) implies the overcoming of a very high free energy barrier of 39.5 kJ·mol^−1^ (see Fig. 4A and Fig. 5A), which makes O_2_ diffusion via this pathway very slow and difficult.

Over the last 20 years, several biochemical studies [20]–[22] have tried to demonstrate that this pathway is the one used for O_2_ diffusion in the A-type C*c*oxs, but, until now, no direct measurement proved this unequivocally. All the tested mutations (e.g V287_I_I [20], [21] and G283_I_V [22]) were located too close to the two metals (Fe in heme *a_3_* and Cu in Cu_B_) in the BNC, which made the interpretation of the results very difficult. It was impossible to unambiguously distinguish between the steric hindrance of the O_2_ channel (introduced by the mutations) and the perturbation in the structure and local binding kinetics at the BNC. Moreover, until now, only one X-ray structure of the A-type C*c*oxs has been pressurized with xenon (considered to be an O_2_ analog) [13]. In this structure [13], two hydrophobic xenon binding sites were identified, none of them located beyond the constriction point of channel 1. One xenon was observed at the entrance of the X-ray inferred channel while the other was close to E286_I_. Since no xenon was observed after the constriction region, there is still no consensus whether this pathway is indeed a functional channel for O_2_ diffusion in these C*c*oxs. Additionally, Hofacker and Schulten [31], with the objective of studying O_2_ diffusion towards the BNC, performed several MD simulations of C*c*oxs with several explicit O_2_ molecules. The authors used the locally enhanced sampling (LES) technique [63], [64] to identify the pathway used by O_2_ both in a bacterial (*P. denitrificans*) and in the bovine C*c*ox enzyme. In their simulations, some of the O_2_ molecules reached the BNC via a unique and well-defined channel located in the same region as the X-ray inferred channel from [13]. Given the very short duration of their simulations (picoseconds), the observation of such events was only possible due to the lowered free energy barriers experienced by the O_2_ molecules when using the LES technique. Nevertheless, a single protein trajectory is still effectively employed in the LES approach, making the protein conformational sampling of that study more limited than the one in the present study. Finally, Farantos *et al.* [32] by performing ILS calculations in the A-type C*c*ox from *P. denitrificans* and in the B-type C*c*ox enzyme from *T thermophilus*, were able to calculate the free energy surfaces for the interaction of several small polar (CO, NO) and apolar (O_2_ and Xe) around the BNC region. Regarding the X-ray inferred channel, their results correlate very well with the ones reported in the present study and the high affinity cavity Xe_1_ observed by the authors in [32] coincides with minima M_11_ in the O_2_ channel 1.

The channel 2 (Fig. 3A, Fig. 4C and 4D) also starts at the membrane region and has only one possible entrance point located between the transmembrane helices 13 and 16 of subunit I, around residues V234_I_, L238_I_ and V325_I_. Moreover, this channel terminates close to Y288_I_, a tyrosine residue covalently bonded to H284_I_, one of the Cu_B_-coordinating histidines. Y288_I_ is located in the end of the K proton-conducting pathway and it has been suggested to be an essential residue for the catalytic mechanism, supplying the proton used in the cleavage of the O-O bond (see [65] for a review). The energy profile for this alternative channel (Fig. 4C and 4D) is characterized by one very low local free energy minimum (M_19_ in Fig. 4C) located between Y288_I_ and T359_I_ and by a high free energy minimum (M_13_ in Fig. 4C) around L292_I_ and I323_I_. The highest energy barriers for this small pathway are 20.4 kJ·mol^−1^ (Fig. 5B), which are substantially smaller than the energy barrier in the constriction point of the O_2_ channel 1 (39.5 kJ·mol^−1^). However, the high energy minimum M_13_ located in the middle of the channel (see Fig. 4C) may hinder O_2_ diffusion via this pathway.

Lastly, the O_2_ channel 3 approaches the BNC from the subunit II side (Fig. 3A, Fig. 4C and 4D) and its entrance is located between the transmembrane helices 28 and 30 of subunit II, around residues F71_II_ and V167_II_. This channel runs parallel to the heme *a_3_* hydroxylethylfarnesyl tail and also terminates just bellow Y288_I_. It is generally formed by hydrophobic and aromatic residues, such as I363_I_, F391_I_ and V272_II_ (see Fig. 4D). The energy profile for this alternative channel (Fig. 4C) is characterized by only one deep local energy minimum (M_19_ in Fig. 4B) located between Y288_I_ and T359_I_ and by several smaller (in comparison with the O_2_ channel 1) energy barriers, which suggest a higher probability of O_2_ passage. In this pathway, the largest energy barriers (19.9–22.3 kJ·mol^−1^ in Fig. 5C) are related with the zone of the hydroxylethylfarnesyl tail of heme *a_3_* and the side chains of I363_I_ and F391_I_. This alternative O_2_ channel was firstly suggested by Tsukihara *et al.* [7], based on the X-ray structure of the bovine heart C*co*x, as one of the three possible O_2_ entrance points in the A-type oxidases. More recently, Farantos *et al.* [32] were also able to observe the final part of this channel (located close to heme *a_3_*) in the A-type C*c*ox from *P. denitrificans*.

Even though only subunits I and II were considered in this work, the entry points for all the channels described above are not obstructed by the missing subunits.

Moreover, it is also interesting to notice that all O_2_ channels overlap in space with canonical proton pathways. The O_2_ channel 1, which corresponds to the X-ray inferred channel, overlies with the end of the D-pathway, whereas the two alternative channels (O_2_ channel 2 and channel 3), which end close to Y288_I_, overlap with the final part of the K-pathway.

For the two alternative channels identified here, the O_2_ pathway is not permanently open and by this reason escaped detection when the static X-ray structures were inspected (e.g [7], [12], [13]). These channels resemble a chain of separate, multiple and transiently formed hydrophobic cavities that are interconnected with each other via the fluctuations of the residues lining the pathway. During the diffusion process, O_2_ enters the protein and probably due to the thermal fluctuations of the heme *a_3_* tail and residues lining the pathways, it is allowed to diffuse further into the protein core until it reaches the BNC.

Deciphering the actual role of these O_2_ channels will require further mutational and biochemical analysis. Several mutations sites can be suggested (see Table 1), on the three O_2_ channels, in order to clarify the role of each pathway in O_2_ diffusion towards the BNC in the A-type C*c*oxs.

**Table 1:** Point mutations suggested for the three putative O_2_ channels.

Residue	Location	Suggested Mutations
W172_I_	Channel 1	less bulkier side-chain residues (e.g. Y or F)
F282_I_	Channel 1	less bulkier side-chain residues (e.g. T, V, A, G)
L292_I_	Channel 2	longer and bulkier side-chain residues (e.g. M or F)
I323_I_	Channel 2	longer and bulkier side-chain residues (e.g. M or F)
I363_I_	Channel 3	longer side-chain residues (e.g. L or M)
V72_II_	Channel 3	longer side-chain residues (e.g. L or M)
V107_II_	Channel 3	bulkier side-chain residue (e.g. F)

For the X-ray inferred channel, it is clear that alternative mutation sites, located farther away from the metal ions in the BNC, need to be constructed and studied in order to clarify the actual role of this pathway in O_2_ diffusion. W156_I_ and F282_I_ are excellent candidates for mutation experiments and their mutation for less bulkier residues, such as tyrosine and threonine (similar to what is observed in the *T. thermophilus ba_3_* enzyme [23]–[25]) would give important insights into how O_2_ diffusion occurs via this channel. Furthermore, for the alternative channels, the residues lining both pathways are also good candidates for mutational studies, because longer and/or bulkier side-chains could, in principle, obstruct the alternative channels and thus clarify the question of whether these channel are, indeed, used to supply O_2_ to the BNC in the A-type C*c*oxs. Nevertheless, until new experimental data is available, we cannot rule out the hypothesis that all three channels may be working under physiological conditions.

### Concluding remarks

Although A-type C*c*oxs have been widely studied during the last four decades, the details of the O_2_ diffusion mechanism are still very incomplete. In particular, the existence and the characteristics of the channel(s) used by O_2_ to travel from the solvent/membrane to the BNC are still unclear. In this study, we have used an integrated strategy of all-atom MD simulations (with and without explicit O_2_ molecules) and ILS calculations, designed to examine and characterize the O_2_ delivery channels in fully reduced C*c*ox from *R. sphaeroides*. Altogether, our results suggest that O_2_ does not diffuse unspecifically inside this protein and instead, uses three well-defined channels running from the interior of the membrane (where O_2_ solubility is higher than in the aqueous phase) towards the C*c*ox core. The first pathway has two entrance points, located between helices 5 and 8 and helices 11 and 13 of subunit I, which converges into the constriction point just before the BNC. This channel correlates very well with the channel inferred from the available X-ray structures. The second pathway has only one entry located between the transmembrane helices 13 and 16 of subunit I and it terminates close to Y288_I_. The third identified pathway approaches the BNC from the subunit II side. This channel runs parallel to the heme *a_3_* hydroxylethylfarnesyl tail and also terminates just below Y288_I_. According to our observations, the hydrophobic channel detected in the X-ray structures does not constitute the most likely (energetically preferred) entrance point for the O_2_ molecules in this C*c*ox. From the O_2_ affinity map, O_2_ accesses the BNC via the alternative dynamic channels formed by transient hydrophobic cavities, whose opening and closure is regulated by the thermal fluctuations of the protein. This may be the reason why these channels were not visible in the static X-ray structures.

In summary, our results suggest that the original hypothesis (based on static X-ray structures and mutational studies on A-type C*c*ox) that proposed, that O_2_ permeation occurs via a unique, continuous and permanently open channel, is indeed a simplification. Our current work does not rule out the role of the X-ray inferred channel, but suggests other alternative routes to the BNC. Furthermore, it emphasizes the need to take into account the dynamic behavior of the protein in order to obtain a more complete description of the O_2_ putative channels and a more detailed picture of the mechanisms underlying O_2_ diffusion in these C*c*oxs.

## Materials and Methods

### Starting structure

The 2.15 Å resolution crystal structure of the fully reduced C*c*ox from *R. sphaeroides* (pdb code: 3FYE) [15] was used as the starting point for this work. This X-ray structure only contains the minimum functional unit (subunits I and II) for C*c*ox. Only the water molecules with a relative accessibility to the solvent lower than 50% were kept. The relative accessibility of water was computed using the program ASC [66], [67], resulting in the selection of 240 water molecules.

Since the GROMOS 54A7 force-field [68] lacks the proper parameterization for the C*c*ox redox centers, the atomic partial charges for reduced Cu_A_, heme *a* and BNC centers were calculated using quantum mechanical calculations with the software Gaussian09 [69] and RESP fitting [70], as described in detail in S1 Text in section 1. The van der Waals parameters for the iron atom (located in the two heme groups) were taken from the universal force field [71] whereas the remaining bonded and van der Waals parameters for the metal centers were adapted from the GROMOS 54A7 force field [68].

The protonation state of each individual protonable group at pH 7.0 was determined using a combination of Poisson-Boltzmann calculations, performed with the package MEAD (version 2.2.5) [72]–[74], and Metropolis Monte Carlo simulations, using the program PETIT (version 1.3) [75]. These calculations were performed using the methodologies described in [75], [76]. For details related with the determination of the protonation state of the protonatable residues, see section 2 in S1 Text.

### C*c*ox insertion into a lipid membrane

Subunits I and II of C*c*ox were inserted in a pre-equilibrated dimysristoylphosphatidylcholine (DMPC) lipid membrane (for details related with the membrane construction, equilibration and characterization see [77]). The optimal position of the protein relative to the membrane was determined using the location of the charged residues in the transmembrane helices as a reference. After C*c*ox insertion into the membrane, all the DMPC molecules located within a cut-off distance of 1.2 Å from the protein atoms were removed, as described in detail elsewhere [77], [78]. Subsequently, the system (protein, membrane and crystallographic waters) was hydrated in a orthorhombic box using a pre-equilibrated box of SPC water molecules [79]. The water molecules misplaced in the center of the membrane (formed by the highly hydrophobic lipid tails), were removed upon visual inspection. The final system contained the reduced C*c*ox embedded in a 175 DMPC lipid membrane surrounded by 19,645 water molecules, in a total of 75,178 atoms.

### MD simulations of C*c*ox inserted in a lipid membrane

All MD simulations were performed using the software package GROMACS 4.0.4 [80] together with the united atom GROMOS 54A7 force-field [68] for the protein and lipids and the previously described atomic partial charges and parameters for the redox centers. The simple point charge (SPC) water model was used [79]. Periodic boundary conditions were applied to all simulations. Non-bonded interactions were calculated using a twin range method [81] with short and long-range cut-offs of 8 and 14 Å, respectively. A reaction field correction [82], [83] was applied for the truncated electrostatic interactions, considering a dielectric constant of 62 [84]. The SETTLE algorithm [85] was used to constraint the bond lengths and angle in water molecules, while the LINCS algorithm [86] was used to keep all remaining bonds constrained. The time step for integrating the equations of motion was 0.002 ps and the neighbor list was updated every 5 steps. The simulations were performed at the constant temperature of 310 K, which is above the phase transition temperature for the DMPC lipids (T_m_ = 296–297 K) in order to ensure that the membrane is in the liquid crystalline state [87]. A Berendsen heat bath [88] was used, with separate couplings for the protein, membrane and solvent, using a relaxation time constant of 0.1 ps. The pressure was coupled semi-isotropically (coupling constant of 5.0 ps and isothermal compressibility of 4.6×10^−5^ bar^−1^ [84]), resulting in an independent coupling of the lateral (P_x+y_) and perpendicular (P_z_) pressures. For all simulations, the x+y and z pressure components were kept at 1 atm and no surface tension was applied [84]. These simulation conditions were shown by Poger *et al*. [84], [89] to correctly reproduce several experimental measurements for this type of membranes.

The system was energy minimized with the steepest-descent method in order to remove excessive strain by performing 5000 steps of steepest-descent minimization with harmonic restraints applied to all non-hydrogen atoms (protein and lipids), followed by further 5000 steps restraining the non-hydrogen atoms of the protein, ending with 5000 steps with restraints applied to the Cα atoms only. After the minimization procedure, and in order to allow proper repacking of the lipids around the protein, a 20 ns MD relaxation was executed in three steps. First, a 0.5 ns simulation was performed with position restraints to all non-hydrogen atoms of the protein and solvent, at constant temperature and pressure. Afterwards, an additional 0.5 ns simulation was performed, with position restraints applied to the non-hydrogen atoms of the protein only. Finally, only the C_α_ atoms were restrained for a period of 19 ns. A force constant of 1000 kJ mol^−1^nm^−2^ was used for all the steps that included harmonic position restraints. The unrestrained simulations started after these 20 ns of restrained simulation.

In order to reduce the well known sampling problems in membrane-protein simulations, five MD simulations, 100 ns each, were performed, resulting in 0.5 µs of total simulation time. All replicates were initiated with different sets of random velocities. These simulations will be hereafter designated as O_2_-free simulations.

### MD simulations of C*c*ox inserted in a lipid membrane with explicit O_2_

After 20 ns of restrained simulations, we randomly added 84 molecules of dioxygen (O_2_) in the solvent zone of each system. No O_2_ was placed inside the protein nor inside the hydrophobic core of the membrane (see S4 Figure in S1 Text). This new set of simulations will be, hereafter, designated as O_2_ simulations. The water molecules within a 2 Å distance from the O_2_ molecules were deleted, similarly to the procedure described in [33].

In order to allow the solvent to adapt to the newly added O_2_ molecules, a 0.5 ns MD simulation with position restraints on all non-hydrogen atoms (force constant of 1000 kJ mol^−1^nm^−2^) was performed. After this initialization procedure, unrestrained MD simulations were carried out and the simulation conditions and parameters were similar to the ones described previously for the MD simulations without O_2_, except for the temperature coupling groups used. In this set of simulations, the O_2_ molecules were included in the same group as the protein. 5 MD simulations, 100 ns each, were performed. The parameters for the O_2_ molecules were taken from the previously published work of Victor *et al* [90].

The 84 O_2_ molecules added to the system corresponds to an O_2_ concentration of ∼0.235 M, which is higher than the experimental solubility of this gas in water. However, this high O_2_ concentration does not affect the structural properties of the protein as shown in S5 Figure in S1 Text.

Moreover, the use of this high number of O_2_ molecules is necessary to obtain reliable statistics within a reasonable simulation time.

### Implicit ligand sampling (ILS) calculations in C*c*ox

Sites with high O_2_ affinity were determined using the ILS method, as previously described in [29]. In this method, the potential of mean force for placing an O_2_ molecule in any position inside the protein is calculated according to:
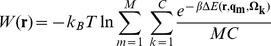
(2)


In equation 2, the implicit ligand potential of mean force, 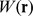, is an average over a finite number of protein and solvent configurations (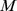) and over a number of different equally probable orientations of the ligand (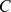). Moreover, 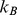 is the Boltzmann constant, 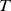 is the absolute temperature, 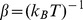 and 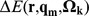 is the interaction energy between the protein and solvent configuration (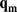) with the ligand located at position 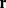 with the orientation 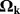.

In our case, the O_2_ free energy map was constructed using the last 50 ns (for each replicate) of the O_2_-free simulations. For the calculations, all 50005 conformations (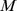  = 10001 conformations x 5 replicates in equation 2) were fitted to the X-ray structure using the C_α_ atoms. A grid of 51×55×87 dimensions was used with a grid spacing of 1 Å and 400 O_2_ insertions were performed per grid point (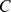  = 400 in equation 2). All calculations were carried out using a version of GROMACS 4.0.4 Widom TPI algorithm, modified almost in the same way as described in [33]. The only difference is that the ligand insertions here were made within the whole space of the grid cube (the grid cube is centered at the insertion point and with edge length equal to the grid spacing), while in the previous work (described in [33]) the insertions were only possible within the inscribed sphere on the grid cube.

The 3D free energy map obtained describes the Gibbs free energy of moving an O_2_ molecule from vacuum to a given position in the system, Δ*G*_vac→prot_(O_2_). This map was then converted into the Δ*G*_wat→prot_(O_2_) map of interest using a Δ*G*_wat→prot_(O_2_) calculated as described in [33].

### Data analysis

The secondary structure assignment was performed with the program DSSP [91]. To determine the percentage of secondary structure loss relative to the X-ray structure, the secondary structure classes considered were: α-helix, 3_10_-helix, 5-helix, β-sheet and β-bridge.

For the energy landscape analysis, we used the method described in [33]. In short, this method classifies the energy landscape into energy basins through a steepest-descent tessellation and, afterwards, identifies the lowest-energy point within the boundaries between each pair of neighboring basins, i.e. the saddle point between those basins. After this procedure, a network of paths between all energy minima of the landscape can be constructed using the steepest-descent paths from the saddle points to the minima. A cutoff of 20 kJ·mol^−1^ was used for the network construction.

The errors of the free energy profiles were calculated using two blocks: the first block corresponds to the frames ranging from 50 ns to 75 ns (for all five replicates) whereas the second block contains all the frames ranging from 75 ns to 100 ns. The errors were determined as half the difference of the energies observed between the two blocks for each minina and for each transition. The method used for error calculation assumes that similar minima and pathways can be identified in the two blocks. However, for the O_2_ channel 1, the pathways connecting M_6_ to M_8_ and M_10_ to M_11_ were not visible in one of the blocks and by this reason their associated errors could not be calculated.

## Supporting Information

S1 Text **Supporting information.** This file includes 6 distinct sections: 1-Parameterization of the redox centers. 2- Protonation state of protonable residues at pH = 7.0. 3- C*c*ox inserted in a lipid membrane with explicit O_2_. 4- C*c*ox conformational drift during the MD simulations. 5- O_2_ diffusion in a DMPC membrane. 6- O_2_ molecules inside C*c*ox.
(PDF)
