# Supplementary material for: Exploring O2 Diffusion in A-Type Cytochrome c Oxidases: Molecular Dynamics Simulations Uncover Two Alternative Channels towards the Binuclear Site
Source: PLoS Comput Biol. 2014 Dec 4;10(12):e1004010. doi: 10.1371/journal.pcbi.1004010 (PMC4256069; doi:10.1371/journal.pcbi.1004010)
Supplement: S1 Text — Supporting information. This file includes 6 distinct sections: 1-Parameterization of the redox centers. 2- Protonation state of protonable residues at pH = 7.0. 3- Ccox inserted in a lipid membrane with explicit O2. 4- Ccox conformational drift during the MD simulations. 5- O2 diffusion in a DMPC membrane. 6- O2 molecules inside Ccox. (PDF) [file pcbi.1004010.s001.pdf]

**Exploring $O_2$ Diffusion in A-type Cytochrome *c* Oxidases:** Molecular Dynamics Simulations Uncover Two Alternative Channels towards the Binuclear Site.

A. Sofia F. Oliveira, João M. Damas, António M. Baptista, Cláudio M. Soares

## Supporting Information

### 1. Parameterization of the redox centers

Since the GROMOS 54A7 force-field [1] lacks the parameterization for the reduced bis-histidine heme *a* group, for the copper center and for the BNC, we had to determine, using quantum chemical (QM) methods, atomic partial charges for the three Ccox's redox centers: $Cu_A$ center (Figure S1), heme *a* (Figure S2) and BNC (Figure S3). For all centers, the models were built based in the reduced structure from R. s*pharoides* (pdb code: 3FYE [2]). These models include the metal atoms and, in general, the side-chains of the centers coordinating residues up to $C_\beta$. The only exception was for the $Cu_A$ center (Figure S1), where the main-chain down to $C_\alpha$ was considered for $E254_{II}$ and $L255_{II}$, and the side-chain down to $C_\gamma$ was used for $M263_{II}$.
In the case of heme groups, both the propionates and the aliphatic tail were not included in the QM calculations and were both substituted by methyl groups (see Figures S2 and S3 for details). All protons were energy optimized using the software GAUSSIAN09 [3] by fixing the positions of all hetero-atoms. The optimizations were performed using B3LYP and the basis sets 6-31G(d) for hetero-atoms and 6-31G(3df) for metal ions. Afterwards, single-point calculations were carried out on the resulting optimized structures using B3LYP and the cc-pVTZ basis sets. The calculations included the solvent effects (using PCM) in a medium of dielectric constant of 4. The electrostatic potentials in space derived were then fitted using the restrained electrostatic potential (RESP) method [4] in order to derive the atomic partial charges for the atoms of interest (united atom approach, with the exception for the polar and aromatic hydrogens). Since the RESP method has a problem when dealing with heavily buried atoms (such as the metal atoms in our centers), the atomic partial charges for the metals were constrained to the value of their Mulliken charge found previously. This procedure avoided unphysically high values for the partial charges like the ones obtained in an unconstrained fitting. The final partial charges for the centers are described in Tables S1, S2 and S3.

The van der Waals parameters for the Fe atom (located in the heme groups) were taken from the universal force field [5], whereas the remaining bonded and van der Waals parameters for the metal centers were adapted from the GROMOS 54A7 force field [1].

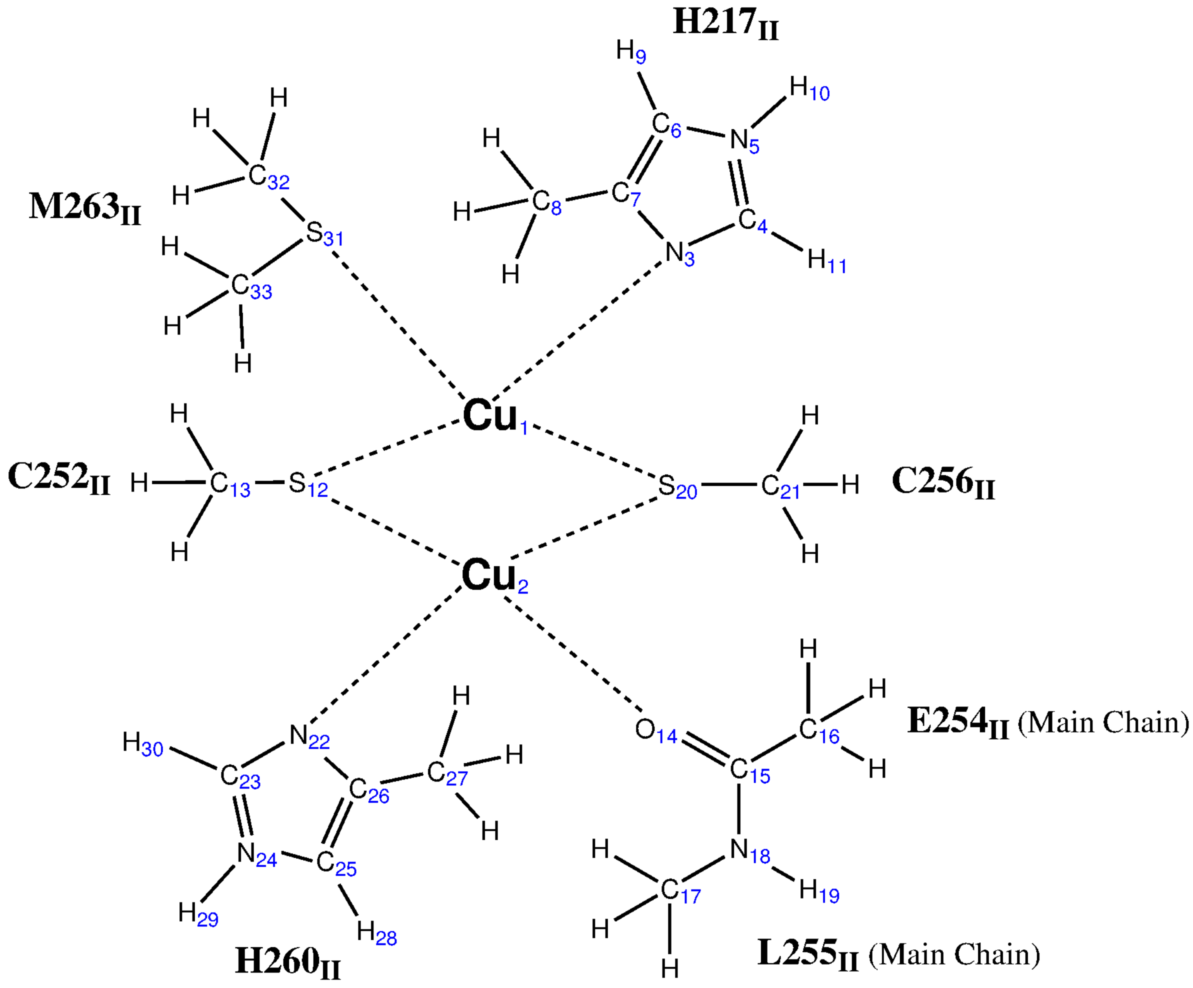


**Figure S1-** Schematic representation of the $Cu_A$ center in the reduced form. In our parameterization only the polar and aromatic hydrogens were considered explicitly.

**Table S1-** Atomic partial charges for the $Cu_A$ center in the reduced state.

| Atom number | Atomic partial charge | Atom number | Atomic partial charge | Atom number | Atomic partial charge |
|---|---|---|---|---|---|

| | |
|---|---|
| $Cu_1$ | 0.300 |
| $Cu_2$ | 0.476 |
| $N_3$ | -0.163 |
| $C_4$ | 0.080 |
| $N_5$ | -0.173 |
| $C_6$ | -0.379 |
| $C_7$ | 0.134 |
| $C_8$ | 0.051 |
| $H_9$ | 0.224 |
| $H_{10}$ | 0.331 |
| $H_{11}$ | 0.101 |

| | |
|---|---|
| $S_{12}$ | -0.653 |
| $C_{13}$ | 0.075 |
| $O_{14}$ | -0.419 |
| $C_{15}$ | 0.408 |
| $C_{16}$ | 0.040 |
| $C_{17}$ | 0.248 |
| $N_{18}$ | -0.520 |
| $H_{19}$ | 0.310 |
| $S_{20}$ | -0.681 |
| $C_{21}$ | 0.071 |
| $N_{22}$ | -0.160 |

| | |
|---|---|
| $C_{23}$ | -0.215 |
| $N_{24}$ | 0.017 |
| $C_{25}$ | -0.416 |
| $C_{26}$ | 0.150 |
| $C_{27}$ | 0.069 |
| $H_{28}$ | 0.221 |
| $H_{29}$ | 0.284 |
| $H_{30}$ | 0.221 |
| $S_{31}$ | -0.400 |
| $C_{32}$ | 0.157 |
| $C_{33}$ | 0.208 |

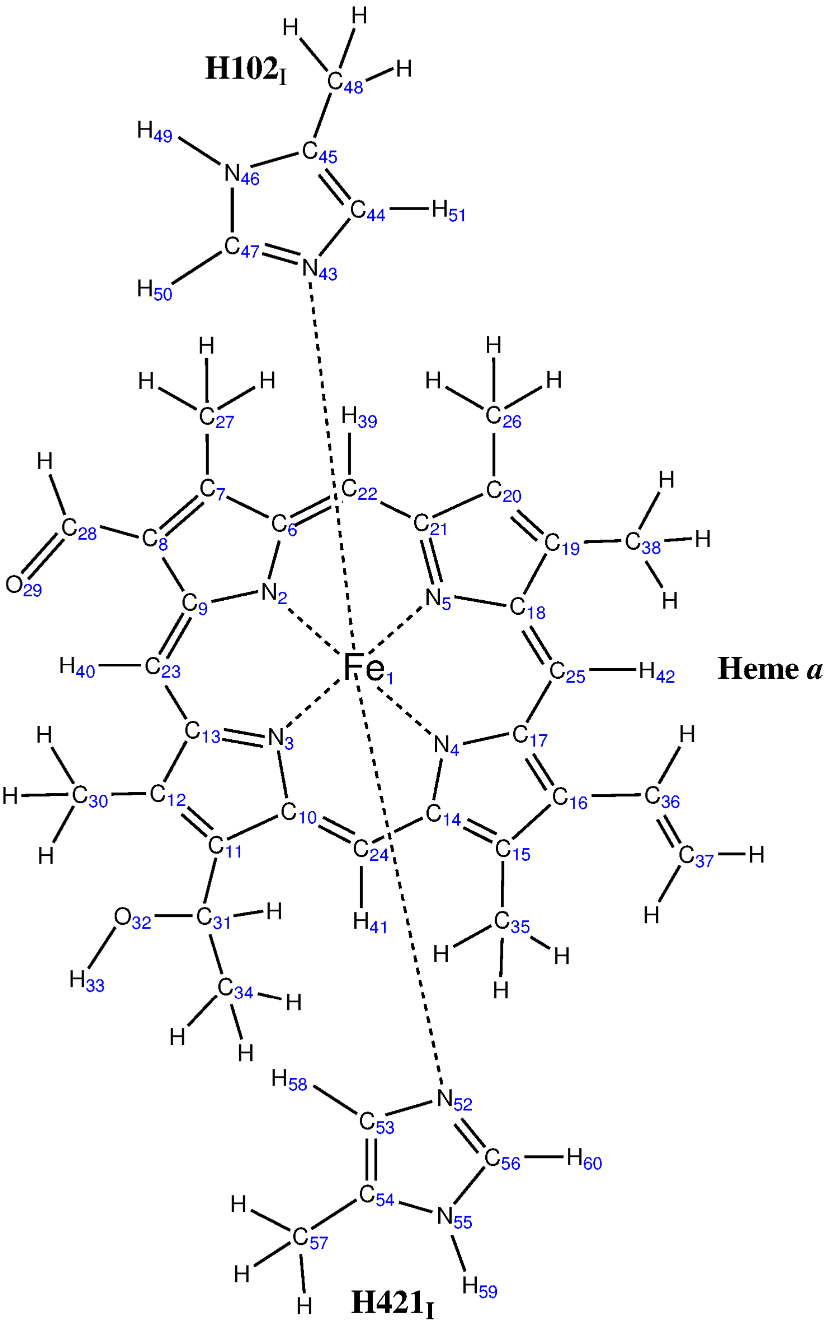


**Figure S2-** Schematic representation of the Heme *a* center in the reduced state.

**Table S2-** Atomic partial charges for the Heme *a* center in the reduced state.

| Atom number | Atomic partial charge | Atom number | Atomic partial charge | Atom number | Atomic partial charge |
|---|---|---|---|---|---|
| $Fe_1$ | 0.673 | $C_{21}$ | 0.066 | $H_{41}$ | 0.139 |

| | | | | | |
|---|---|---|---|---|---|
| $N_2$ | -0.492 | $C_{22}$ | -0.230 | $H_{42}$ | 0.383 |
| $N_3$ | -0.279 | $C_{23}$ | -0.343 | $N_{43}$ | 0.173 |
| $N_4$ | -0.524 | $C_{24}$ | -0.231 | $C_{44}$ | -0.438 |
| $N_5$ | -0.440 | $C_{25}$ | -0.803 | $C_{45}$ | 0.095 |
| $C_6$ | 0.091 | $C_{26}$ | 0.086 | $N_{46}$ | -0.268 |
| $C_7$ | 0.006 | $C_{27}$ | 0.081 | $C_{47}$ | -0.088 |
| $C_8$ | -0.359 | $C_{28}$ | 0.529 | $C_{48}$ | 0.101 |
| $C_9$ | 0.384 | $O_{29}$ | -0.552 | $H_{49}$ | 0.336 |
| $C_{10}$ | 0.080 | $C_{30}$ | 0.053 | $H_{50}$ | 0.185 |
| $C_{11}$ | -0.122 | $C_{31}$ | 0.290 | $H_{51}$ | 0.269 |
| $C_{12}$ | 0.042 | $O_{32}$ | -0.630 | $N_{52}$ | 0.152 |
| $C_{13}$ | 0.027 | $H_{33}$ | 0.396 | $C_{53}$ | -0.501 |
| $C_{14}$ | -0.052 | $C_{34}$ | -0.016 | $C_{54}$ | 0.130 |
| $C_{15}$ | 0.109 | $C_{35}$ | 0.086 | $N_{55}$ | -0.317 |
| $C_{16}$ | -0.468 | $C_{36}$ | -0.069 | $C_{56}$ | -0.011 |
| $C_{17}$ | 0.749 | $C_{37}$ | 0.061 | $C_{57}$ | 0.097 |
| $C_{18}$ | 0.353 | $C_{38}$ | 0.073 | $H_{58}$ | 0.298 |
| $C_{19}$ | -0.146 | $H_{39}$ | 0.146 | $H_{59}$ | 0.347 |
| $C_{20}$ | -0.085 | $H_{40}$ | 0.215 | $H_{60}$ | 0.162 |

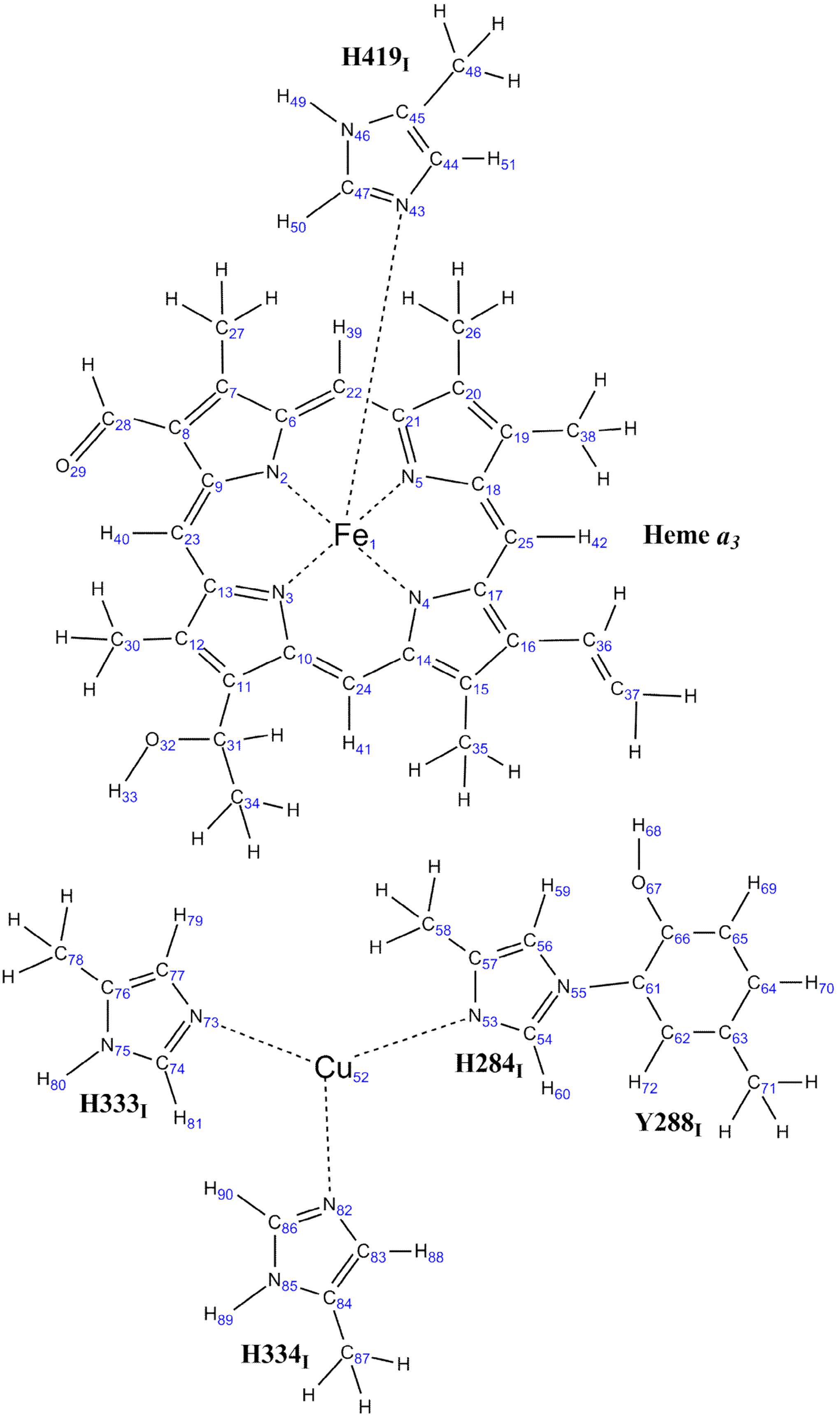


**Figure S3-** Schematic representation of the BNC center in the reduced state.

**Table S3-** Atomic partial charges for the BNC center in the reduced state.

| Atom number | Atomic partial charge |
|---|---|
| $Fe_1$ | 1.046 |
| $N_2$ | -0.644 |
| $N_3$ | -0.531 |
| $N_4$ | -0.796 |
| $N_5$ | -0.727 |
| $C_6$ | 0.259 |
| $C_7$ | -0.041 |
| $C_8$ | -0.355 |
| $C_9$ | 0.524 |
| $C_{10}$ | 0.462 |
| $C_{11}$ | -0.246 |
| $C_{12}$ | -0.071 |
| $C_{13}$ | 0.236 |
| $C_{14}$ | 0.360 |
| $C_{15}$ | 0.146 |
| $C_{16}$ | -0.593 |
| $C_{17}$ | 0.714 |
| $C_{18}$ | 0.459 |
| $C_{19}$ | -0.161 |
| $C_{20}$ | -0.241 |
| $C_{21}$ | 0.471 |
| $C_{22}$ | -0.350 |
| $C_{23}$ | -0.450 |
| $C_{24}$ | -0.433 |
| $C_{25}$ | -0.572 |
| $C_{26}$ | 0.126 |
| $C_{27}$ | 0.105 |
| $C_{28}$ | 0.504 |
| $O_{29}$ | -0.480 |

| Atom number | Atomic partial charge |
|---|---|
| $C_{31}$ | 0.365 |
| $O_{32}$ | -0.593 |
| $H_{33}$ | 0.365 |
| $C_{34}$ | -0.023 |
| $C_{35}$ | 0.022 |
| $C_{36}$ | 0.159 |
| $C_{37}$ | 0.023 |
| $C_{38}$ | 0.131 |
| $H_{39}$ | 0.134 |
| $H_{40}$ | 0.197 |
| $H_{41}$ | 0.142 |
| $H_{42}$ | 0.183 |
| $N_{43}$ | -0.282 |
| $C_{44}$ | -0.141 |
| $C_{45}$ | -0.024 |
| $N_{46}$ | -0.179 |
| $C_{47}$ | 0.062 |
| $C_{48}$ | 0.138 |
| $H_{49}$ | 0.325 |
| $H_{50}$ | 0.106 |
| $H_{51}$ | 0.159 |
| $Cu_{52}$ | 0.470 |
| $N_{53}$ | -0.560 |
| $C_{54}$ | 0.212 |
| $N_{55}$ | 0.132 |
| $C_{56}$ | -0.142 |
| $C_{57}$ | 0.032 |
| $C_{58}$ | 0.142 |
| $H_{59}$ | 0.163 |

| Atom number | Atomic partial charge |
|---|---|
| $C_{61}$ | 0.000 |
| $C_{62}$ | -0.140 |
| $C_{63}$ | 0.000 |
| $C_{64}$ | -0.140 |
| $C_{65}$ | -0.140 |
| $C_{66}$ | 0.203 |
| $O_{67}$ | -0.611 |
| $H_{68}$ | 0.408 |
| $H_{69}$ | 0.140 |
| $H_{70}$ | 0.140 |
| $C_{71}$ | 0.000 |
| $H_{72}$ | 0.140 |
| $N_{73}$ | -0.272 |
| $C_{74}$ | 0.048 |
| $N_{75}$ | -0.175 |
| $C_{76}$ | -0.059 |
| $C_{77}$ | -0.064 |
| $C_{78}$ | 0.124 |
| $H_{79}$ | 0.124 |
| $H_{80}$ | 0.325 |
| $H_{81}$ | 0.150 |
| $N_{82}$ | -0.438 |
| $C_{83}$ | -0.097 |
| $C_{84}$ | -0.036 |
| $N_{85}$ | -0.028 |
| $C_{86}$ | 0.122 |
| $C_{87}$ | 0.142 |
| $H_{88}$ | 0.163 |
| $H_{89}$ | 0.218 |

| | | | | | | | |
|---|---|---|---|---|---|---|---|
| C30 | 0.092 | | $H_{60}$ | 0.139 | | $H_{90}$ | 0.084 |

## 2. Protonation state of protonable residues at pH=7.0

The protonation state of each individual protonable group in the protein at a given value of pH (in this case pH=7.0) has to be specified prior to the MD simulations. He have determined the protonation states of the protonable residues in C*c*ox using methodologies for studying the thermodynamics of proton binding described in detail in [6,7]. These methodologies use a combination of Poisson-Boltzmann (PB) calculations, performed with the program MEAD (version 2.2.5) [8-10], and Metropolis Monte Carlo (MC) simulations, using the program PETIT (version 1.3) [6]. The GROMOS 54A7 charge set [1] was used for the normal residues, whereas our calculated atomic charges were used for the redox centers**.** The dielectric constants used for the solvent ($\varepsilon_{Sol}$) and for the protein ($\varepsilon_{protein}$) were 80 and 10, respectively [11]. An implicit membrane was introduced in the x-y plane and modeled as a low dielectric ($\varepsilon_{memb}$= $\varepsilon_{prot}$) slab of 40 Å. The PB/MC calculations were done at a fixed redox state (fully reduced), and with steps of 0.2 pH units. The N- and C-termini of both subunits I and II were allowed to titrate. Based on the titration curves, most of the glutamic and aspartic acid residues, propionates and C-termini were found to be negatively charged, whereas most of the lysine and arginine residues and N-termini were found to be positively charged. Five exceptions were observed, namely $K74_I$, $K438_I$, $E286_I$, $D407_I$ and $K362_I$, which were found to have a total charge of zero. $E286_I$, $D407_I$ and $K362_I$ are residues located in the interior of the protein and thus were left in the predicted neutral state. In contrast, $K74_I$ and $K438_I$ are positioned close to the water/membrane interface, where they can easily change their conformation and directly interact with the solvent, and thus were considered to be positively charged. Histidine residues were found to be in different protonation states (Table S4). It should be noted that the histidine residues coordinating the redox centers were not allowed to titrate.

$Y288_I$ is a tyrosine residue that is cross-linked to one of the BNC histidines via its $N\varepsilon_2$ atom. This chemical modification is known to alter the phenol p$K_{mod}$ (the p$K_a$ of the group when it is completely solvated and in the absence of other interactions) [12]. A procedure similar to the one describe in [13] was used for the parameterization of the p$K_{mod}$ of this cross-linked tyrosine residue. The p$K_{mod}$ value that mimic the experimental titration [12] was 8.599 for $\varepsilon_{protein}$=10. These values were calculated considering that $Y288_I$ has two possible tautomers [6].

**Table S4-** Protonation state of C*c*ox free histidines. HisA refers to the neutral residue protonated at the $N_\delta$ position, while HisB corresponds to a neutral residue protonated at $N_\varepsilon$. The HisH represents the protonated histidine both at $N_\delta$ and $N_\varepsilon$ and with the overall charge of +1.

H102$_{I}$, H333$_{I}$, H334$_{I}$, H419$_{I}$, 421$_{I}$, H217$_{II}$ and H260$_{II}$ are coordinating the redox centers (see Figure S1, S2 and S3) and were not allowed to titrate in our PB/MC calculations. H284$_{I}$ is coordinating the $Cu_B$ copper ion and is cross-linked to Y288$_{I}$ and by this reason it is not protonated in $N_\delta$ nor in $N_\varepsilon$.

| Residue | Subunit | Protonation State |
|---|---|---|
| 26 | I | HISA |
| 67 | I | HISH |
| 93 | I | HISB |
| 102 | I | HISA |
| 127 | I | HISB |
| 195 | I | HISB |
| 223 | I | HISA |
| 277 | I | HISB |
| 284 | I | n.a |
| 300 | I | HISA |
| 333 | I | HISA |
| 334 | I | HISA |
| 411 | I | HISA |
| 419 | I | HISA |
| 421 | I | HISA |
| 456 | I | HISB |
| 472 | I | HISA |
| 534 | I | HISB |
| 549 | I | HISB |
| 55 | II | HISB |
| 84 | II | HISB |
| 96 | II | HISA |
| 217 | II | HISB |
| 260 | II | HISB |
| 282 | II | HISH |
| 283 | II | HISH |
| 284 | II | HISH |
| 285 | II | HISH |

## 3. C*c*ox inserted in a lipid membrane with explicit $O_2$

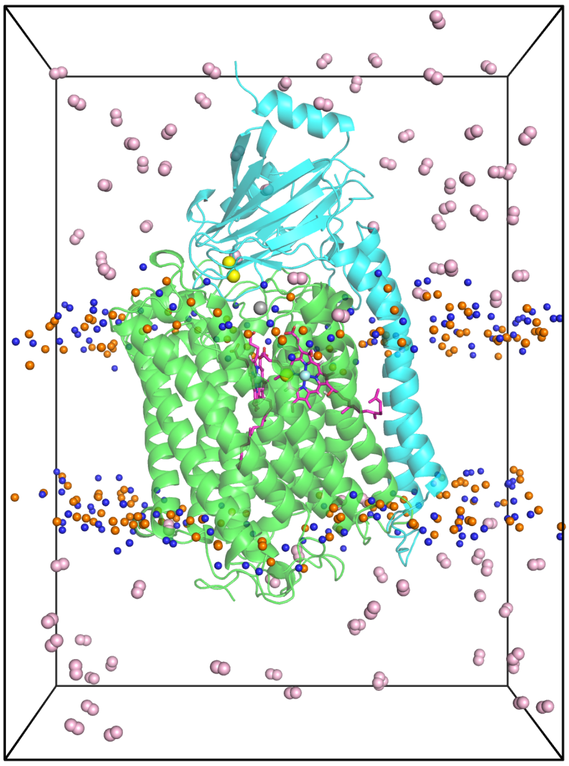

**Figure S4**- Side view of the simulated system with explicit $O_2$ in the beginning of the simulation for one replicate. All $O_2$ molecules (pink spheres) were initially randomly placed in the solvent. The protein is rendered as a carton while the nitrogen and the phosphate atoms from the lipid head groups are represented as blue and orange spheres.

## 4. C*c*ox conformational drift during the MD simulations

In order to study $O_2$ diffusion into C*c*ox, we simulated the protein inserted into a DMPC membrane together with 84 $O_2$ molecules. These simulations are called "$O_2$ simulations". In order to reduce the sampling problems, 5 replicas, each 100 ns long, were simulated. At the beginning of the simulations, all $O_2$ molecules were placed in the solvent.

In addition, and as a control, we have also simulated C*c*ox inserted into a DMPC membrane without $O_2$ (named "$O_2$-free simulations"). For this set, 5 replicates were performed, each 100 ns long.

The behavior of C*c*ox was carefully examined during the 100 ns for the two sets of simulations, by visual inspection and by monitoring several system properties (Figure S5), such as the root mean square deviation from the X-ray structure (Figure S5A and S5B), the secondary structure content (Figure S5C and S5D) and the solvent accessible surface (Figure S5E and S5F). As can be seen in Figure S5, no significant differences were found between the two set of simulations, which indicate that the high concentration of $O_2$ used in our simulations does not affect the conformational stability of C*c*ox.

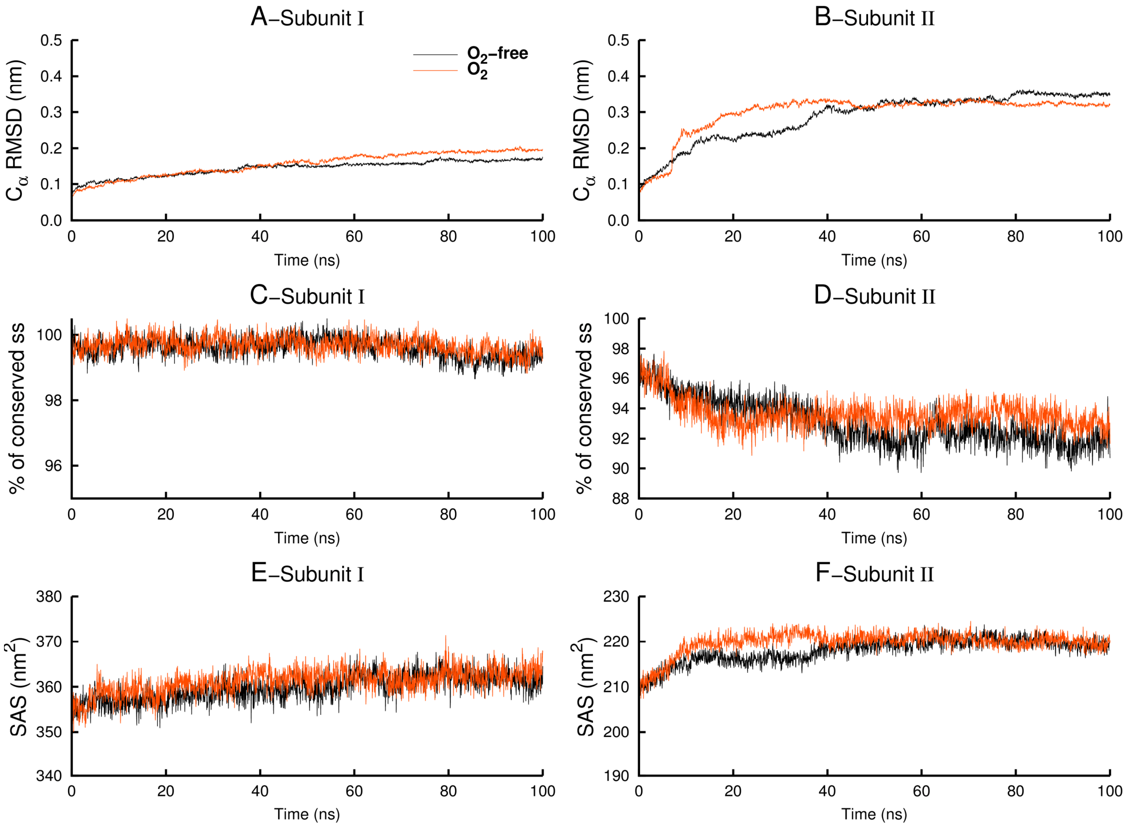

**Figure S5**- Comparison between the simulations with (orange line) and without (black line) $O_2$. **A** and **B**- Average (over the five replicates) $C_\alpha$ RMSD evolution along the simulation time for subunit I (left side) and II (right side). The $C_\alpha$ RMSD was calculated relative to the X-ray structure and was determined after fitting each subunit separately. Due to a high amplitude rigid body motion of helix $31_{II}$ (which will be discussed in the following paragraph), the RMSD was calculated disregarding that region. **C** and **D**- Percentage of residues with conserved secondary structure relative to the X-ray structure (calculated using DSSP [14]) for subunit I and II. The regular secondary structures considered for this measure were the α-helix, the β-sheet, the $3_{10}$ helix and the β-bridge (DSSP classification [14]). **E** and **F**- Time evolution of the Solvent Accessible Surface (SAS) for subunit I and II. All the averages represented in this figure were obtained over all 5 replicates.

In the two sets of simulations, we observed a large movement of helix $31_{II}$ towards the membrane region (see an illustrative example of this movement in Figure S6).

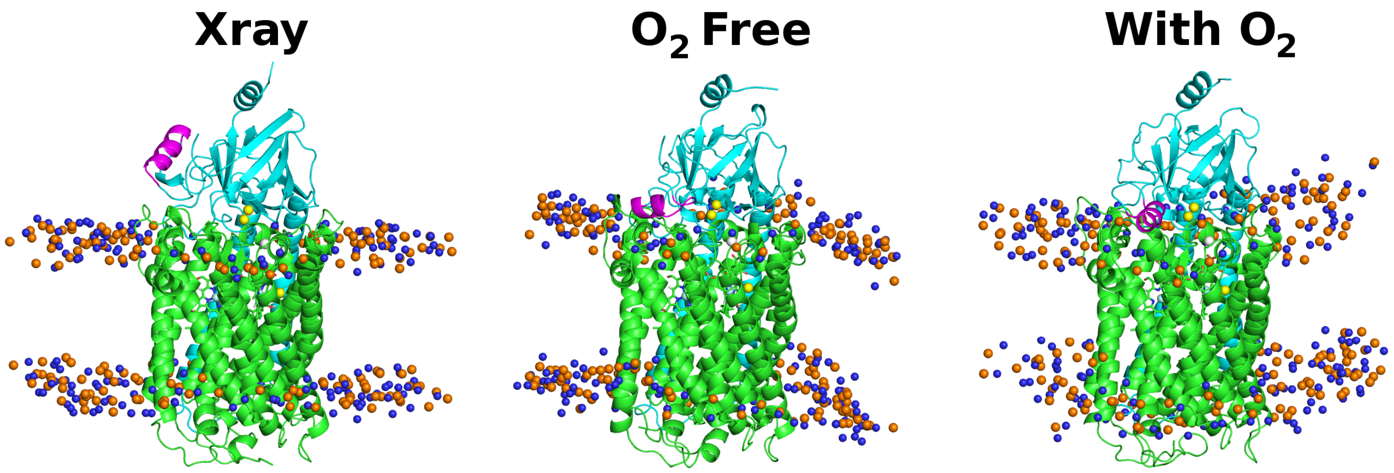


**Figure S6-** Representative example of the helix $31_{II}$ movement in the $O_2$-free and in the $O_2$ simulations. The left side image shows the X-ray structure, while the middle and the right images represent the protein structure (for one of the five replicates) obtained after 100 ns of simulation for the $O_2$ free (middle) and for the $O_2$ simulations (right side). In this image, subunits I and II are colored in green and cyan, respectively. Helix $31_{II}$, is highlighted in magenta. The yellow spheres represent the Cu atoms, whereas the N and P atoms from the lipid head groups are represented as blue and orange spheres. The rest of the lipid atoms are omitted for clarity.

This large movement resulted in a high RMSD for the helix $31_{II}$ region (see Figure S7). In some of the replicates, this rotation started early in the simulations (in the first ten nanoseconds) and it was fast (e.g replicate 2 and 4 in the $O_2$ free and in the $O_2$ simulations, respectively), while in other replicates the rotation started later in the

simulations (after several tens of nanoseconds) and it consisted in a mild and progressive movement (e.g replicate 3 and 4 of the $O_2$ free simulations and replicate 3 in the $O_2$ simulations). Nevertheless, and despite this large amplitude movement, the overall C*c*ox tri-dimensional fold was maintained intact during the 100 ns of simulation, as can be seen by the small percentage of secondary structure loss (<10%) and by the low RMSD values measured (Figure S5).

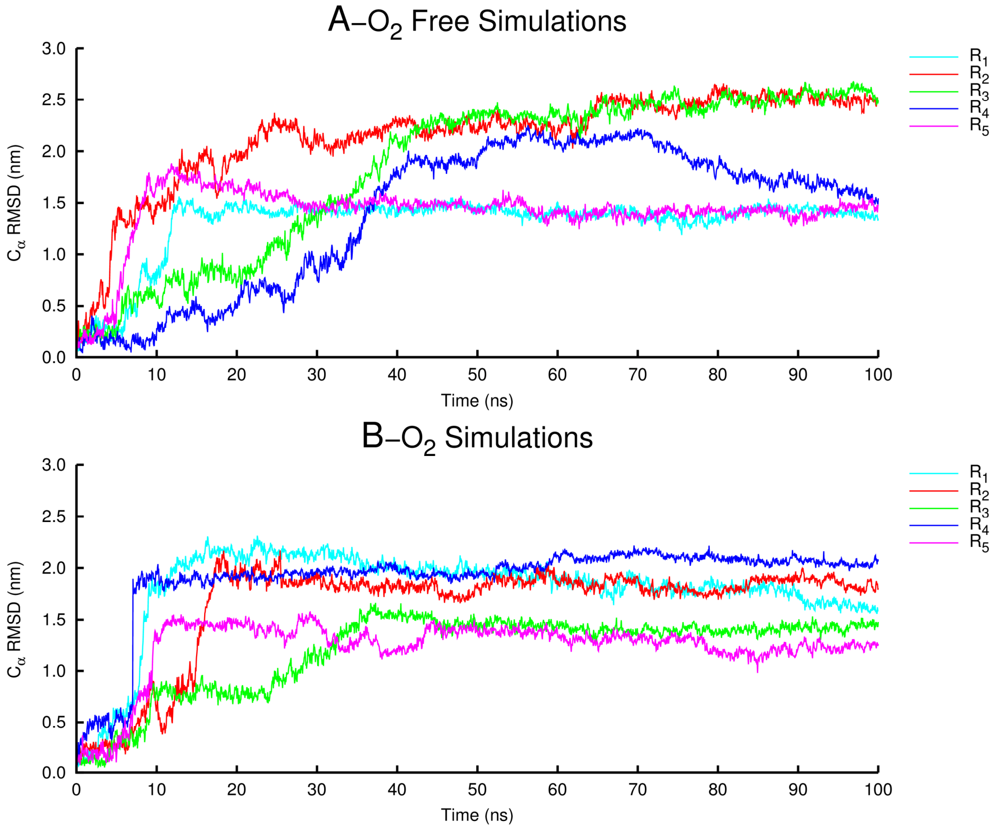


**Figure S7**- Time evolution of the $C_\alpha$ RMSD of helix $31_{\mathrm{II}}$ with respect to its position in the crystal structure, after fitting to the subunit II $C_\alpha$ atoms, for the (**A**) $O_2$ free and the (**B**) $O_2$ simulations.

Helix $31_{\mathrm{II}}$ is a small α-helix formed by 10 residues and is located in the water-soluble globular domain of subunit II. Upon rotation, some of the helix $31_{\mathrm{II}}$ residues establish new interactions (such as H-bonds and van der Waals interactions) with the polar phosphocholine lipid headgroups (Figure S6). This new observed conformation for helix $31_{\mathrm{II}}$ and its direct interactions with the membrane is not present, at least to our knowledge, in any of the crystallographic structures available for the A-type C*c*oxs (e.g. [2,15-21] ) nor has been previously reported in any of the MD studies using C*c*ox (e.g. [22-39]). In the majority of the computational studies available, no membrane was simulated and/or spatial restrictions were imposed on the protein in order to maintain

its structural integrity and avoid severe deviations from the X-ray structures (like in [22,24,28,29,32-36]). In the cases were a lipid membrane was explicitly simulated, sometimes the simulated time was smaller than the time needed to observe such a movement (e.g. [23,25-27,30,31,37]) and/or the simulation conditions were different from the ones used in this work (force-fields, lipids used to built the membrane, X-ray structure used as a starting structures for the simulations), like in [38,39].

We consider that the high amplitude conformational change observed for helix $31_{II}$ is a natural adaptation of the protein to the membrane moiety, which may be different from the crystallographic environment. By the analysis of the crystallographic contacts of helix $31_{II}$, we were able to see that this helix interacts strongly with its symmetry mates in the crystal. By this reason, the conformation for helix $31_{II}$ observed in the X-ray crystals may be strongly influenced by these contacts and may not reflect the real situation when the protein is inserted in a lipid membrane.

## 5. $O_2$ diffusion in a DMPC membrane

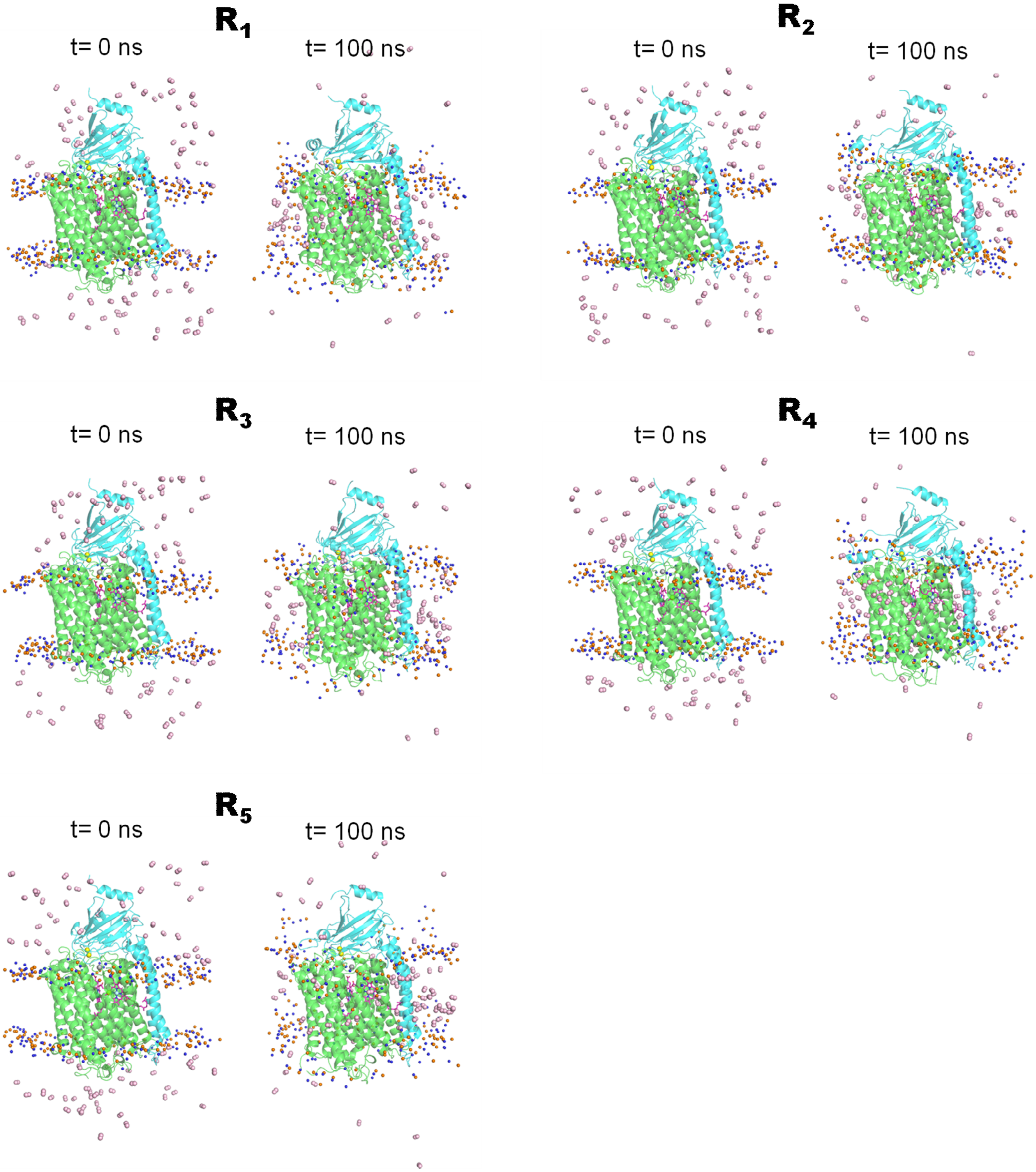


**Figure S8**- Snapshots of the simulated system (C*c*ox, membrane and $O_2$) in the beginning (t=0 ns) and after 100 ns of simulation for the five replicates performed. The protein is rendered as a cartoon while the nitrogen and the phosphorous atoms from the lipid head groups are

represented as blue and orange small spheres. The $O_2$ molecules are highlighted as pink spheres. For clarity purposes, the solvent molecules were not represented in this image

## 6. $O_2$ molecules inside C*cox*

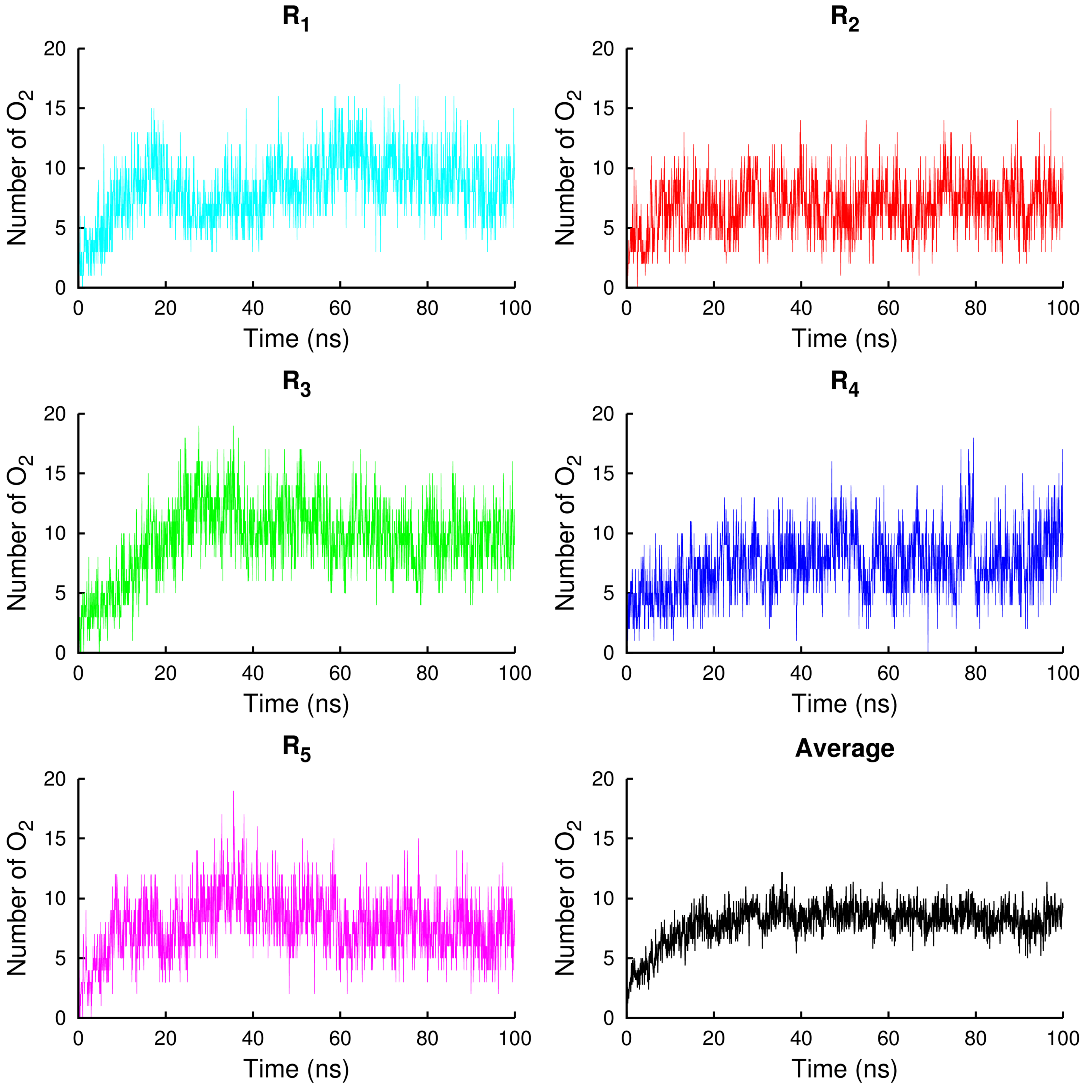


**Figure S9**- Number of $O_2$ molecules inside the protein throughout the simulation time.

## References


1. Schmid N, Eichenberger AP, Choutko A, Riniker S, Winger M, et al. (2011) Definition and testing of the GROMOS force-field versions 54A7 and 54B7. European biophysics journal : EBJ 40: 843-856.
2. Qin L, Liu J, Mills DA, Proshlyakov DA, Hiser C, et al. (2009) Redox-dependent conformational changes in cytochrome C oxidase suggest a gating mechanism for proton uptake. Biochemistry 48: 5121-5130.
3. Frisch MJ, Trucks GW, Schlegel HB, Scuseria GE, Robb MA, et al. (2009) Gaussian 09, Revision A.02. Wallingford, CT: Gaussian, Inc. .

4. Bayly CI, Cieplak P, Cornell WD, Kollman PA (1993) A Well-Behaved Electrostatic Potential Based Method Using Charge Restraints for Deriving Atomic Charges - the Resp Model. J Phys Chem 97: 10269-10280.
5. Rappe AK, Casewit CJ, Colwell KS, Goddard WA, Skiff WM (1992) Uff, a Full Periodic-Table Force-Field for Molecular Mechanics and Molecular-Dynamics Simulations. J Am Chem Soc 114: 10024-10035.
6. Baptista AM, Soares CM (2001) Some theoretical and computational aspects of the inclusion of proton isomerism in the protonation equilibrium of proteins. J Phys Chem 105: 293-309.
7. Teixeira VH, Soares CM, Baptista AM (2002) Studies of the reduction and protonation behavior of tetraheme cytochromes using atomic detail. J Biol Inorg Chem 7: 200-216.
8. Bashford D (1997) An Object-Oriented Programming Suite for Electrostatic Effects in Biological Molecules. In: Ishikawa Y, Oldehoeft RR, Reynders JVW, Tholburn M, editors. Scientific Computing in Object-Oriented Parallel Environments. Berlin: ISCOPE97, Springer. pp. 233-240.
9. Bashford D, Gerwert K (1992) Electrostatic Calculations of the pKa Values of Ionizable Groups in Bacteriorhodopsin. J Mol Biol 224: 473-486.
10. Bashford D, Karplus M (1990) pKa's of ionizable groups in proteins: atomic detail from a continuum electrostatic model. Biochemistry 29: 10219-10225.
11. Teixeira VH, Cunha CA, Machuqueiro M, Oliveira AS, Victor BL, et al. (2005) On the use of different dielectric constants for computing individual and pairwise terms in poisson-boltzmann studies of protein ionization equilibrium. J Phys Chem B 109: 14691-14706.
12. McCauley KM, Vrtis JM, Dupont J, van der Donk WA (2000) Insights into the functional role of the tyrosine-histidine linkage in cytochrome c oxidase. J Am Chem Soc 122: 2403-2404.
13. Soares CM, Baptista AM, Pereira MM, Teixeira M (2004) Investigation of protonatable residues in Rhodothermus marinus caa(3) haem-copper oxygen reductase: comparison with Paracoccus denitrificans aa(3) haem-copper oxygen reductase. J Biol Inorg Chem 9: 124-134.
14. Kabsch W, Sander C (1983) Dictionary of protein secondary structure: pattern recognition of hydrogen-bonded and geometrical features. Biopolymers 22: 2577-2637.
15. Iwata S, Ostermeier C, Ludwig B, Michel H (1995) Structure at 2.8 A resolution of cytochrome c oxidase from Paracoccus denitrificans. Nature 376: 660-669.

16. Tsukihara T, Aoyama H, Yamashita E, Tomizaki T, Yamaguchi H, et al. (1996) The whole structure of the 13-subunit oxidized cytochrome c oxidase at 2.8 angstrom. Science 272: 1136-1144.

17. Yoshikawa S, Shinzawa-Itoh K, Nakashima R, Yaono R, Yamashita E, et al. (1998) Redox-coupled crystal structural changes in bovine heart cytochrome c oxidase. Science 280: 1723-1729.

18. Svensson-Ek M, Abramson J, Larsson G, Tornroth S, Brzezinski P, et al. (2002) The X-ray crystal structures of wild-type and EQ(I-286) mutant cytochrome c oxidases from Rhodobacter sphaeroides. J Mol Biol 321: 329-339.

19. Tsukihara T, Shimokata K, Katayama Y, Shimada H, Muramoto K, et al. (2003) The low-spin heme of cytochrome c oxidase as the driving element of the proton-pumping process. Proc Natl Acad Sci U S A 100: 15304-15309.

20. Muramoto K, Hirata K, Shinzawa-Itoh K, Yoko-O S, Yamashita E, et al. (2007) A histidine residue acting as a controlling site for dioxygen reduction and proton pumping by cytochrome c oxidase. Proc Natl Acad Sci U S A 104: 7881-7886.

21. Koepke J, Olkhova E, Angerer H, Muller H, Peng GH, et al. (2009) High resolution crystal structure of Paracoccus denitrificans cytochrome c oxidase: New insights into the active site and the proton transfer pathways. Biochim Biophys Acta-Bioenergetics 1787: 635-645.

22. Hofacker I, Schulten K (1998) Oxygen and proton pathways in cytochrome c oxidase. Proteins 30: 100-107.

23. Backgren C, Hummer G, Wikstrom M, Puustinen A (2000) Proton translocation by cytochrome c oxidase can take place without the conserved glutamic acid in subunit I. Biochemistry 39: 7863-7867.

24. Wikstrom M, Verkhovsky MI, Hummer G (2003) Water-gated mechanism of proton translocation by cytochrome c oxidase. Biochim Biophys Acta 1604: 61-65.

25. Zheng X, Medvedev DM, Swanson J, Stuchebrukhov AA (2003) Computer simulation of water in cytochrome c oxidase. Biochim Biophys Acta 1557: 99-107.

26. Olkhova E, Hutter MC, Lill MA, Helms V, Michel H (2004) Dynamic water networks in cytochrome C oxidase from Paracoccus denitrificans investigated by molecular dynamics simulations. Biophys J 86: 1873-1889.

27. Cukier RI (2005) A molecular dynamics study of water chain formation in the proton-conducting K channel of cytochrome c oxidase. Biochim Biophys Acta 1706: 134-146.

28. Seibold SA, Mills DA, Ferguson-Miller S, Cukier RI (2005) Water chain formation and possible proton pumping routes in Rhodobacter sphaeroides cytochrome c oxidase: a molecular dynamics comparison of the wild type and R481K mutant. Biochemistry 44: 10475-10485.
29. Xu J, Voth GA (2005) Computer simulation of explicit proton translocation in cytochrome c oxidase: the D-pathway. Proc Natl Acad Sci U S A 102: 6795-6800.
30. Wikstrom M, Ribacka C, Molin M, Laakkonen L, Verkhovsky M, et al. (2005) Gating of proton and water transfer in the respiratory enzyme cytochrome c oxidase. Proc Natl Acad Sci U S A 102: 10478-10481.
31. Tuukkanen A, Kaila VR, Laakkonen L, Hummer G, Wikstrom M (2007) Dynamics of the glutamic acid 242 side chain in cytochrome c oxidase. Biochim Biophys Acta 1767: 1102-1106.
32. Kaila VR, Verkhovsky MI, Hummer G, Wikstrom M (2008) Glutamic acid 242 is a valve in the proton pump of cytochrome c oxidase. P Natl Acad Sci USA 105: 6255-6259.
33. Xu J, Voth GA (2008) Redox-coupled proton pumping in cytochrome c oxidase: further insights from computer simulation. Biochim Biophys Acta 1777: 196-201.
34. Henry RM, Yu CH, Rodinger T, Pomes R (2009) Functional hydration and conformational gating of proton uptake in cytochrome c oxidase. J Mol Biol 387: 1165-1185.
35. Sugitani R, Stuchebrukhov AA (2009) Molecular dynamics simulation of water in cytochrome c oxidase reveals two water exit pathways and the mechanism of transport. Biochim Biophys Acta-Bioenergetics 1787: 1140-1150.
36. Lee HJ, Svahn E, Swanson JM, Lepp H, Voth GA, et al. (2010) Intricate role of water in proton transport through cytochrome c oxidase. J Am Chem Soc 132: 16225-16239.
37. Yang S, Cui Q (2011) Glu-286 rotation and water wire reorientation are unlikely the gating elements for proton pumping in cytochrome C oxidase. Biophys J 101: 61-69.
38. Woelke AL, Galstyan G, Galstyan A, Meyer T, Heberle J, et al. (2013) Exploring the Possible Role of Glu286 in CcO by Electrostatic Energy Computations Combined with Molecular Dynamics. J Phys Chem B 117: 12432-12441.
39. Goyal P, Lu J, Yang S, Gunner MR, Cui Q (2013) Changing hydration level in an internal cavity modulates the proton affinity of a key glutamate in cytochrome c oxidase. Proc Natl Acad Sci U S A 110: 18886-18891.
